# Insights into effective fatigue reducing interventions in kidney transplant candidates: a scoping review

**DOI:** 10.1093/abm/kaaf017

**Published:** 2025-03-14

**Authors:** Avril J Haanstra, Heleen Maring, Yvonne van der Veen, Evelien E Quint, Maya J Schroevers, Adelita V Ranchor, Stefan P Berger, Evelyn J Finnema, Coby Annema

**Affiliations:** Department of Health Sciences, Section of Nursing Science, University of Groningen, University Medical Center Groningen, 9713 GZ Groningen, The Netherlands; Department of Health Sciences, Section of Nursing Science, University of Groningen, University Medical Center Groningen, 9713 GZ Groningen, The Netherlands; Department of Physical Therapy, University of Groningen, University Medical Center Groningen, 9713 GZ Groningen, The Netherlands; Department of Health Sciences, Section of Nursing Science, University of Groningen, University Medical Center Groningen, 9713 GZ Groningen, The Netherlands; Department of Nephrology, Internal Medicine, University of Groningen, University Medical Center Groningen, 9713 GZ Groningen, The Netherlands; Department of Dietetics, University of Groningen, University Medical Center Groningen, 9713 GZ Groningen, The Netherlands; Department of Surgery, University of Groningen, University Medical Center Groningen, 9713 GZ Groningen, The Netherlands; Department of Health Sciences, Section of Health Psychology, University of Groningen, University Medical Center Groningen, 9713 GZ Groningen, The Netherlands; Department of Health Sciences, Section of Health Psychology, University of Groningen, University Medical Center Groningen, 9713 GZ Groningen, The Netherlands; Department of Nephrology, Internal Medicine, University of Groningen, University Medical Center Groningen, 9713 GZ Groningen, The Netherlands; Department of Health Sciences, Section of Nursing Science, University of Groningen, University Medical Center Groningen, 9713 GZ Groningen, The Netherlands; Department of Health Sciences, Section of Nursing Science, University of Groningen, University Medical Center Groningen, 9713 GZ Groningen, The Netherlands

**Keywords:** fatigue, chronic kidney disease, preoperative care, exercise, complementary therapies, psychosocial intervention

## Abstract

**Background:**

Fatigue is a prevalent and debilitating symptom among kidney transplant candidates (KTCs), significantly affecting their quality of life and overall well-being. Its complexity necessitates a comprehensive approach to manage fatigue in this population.

**Purpose:**

To explore the effectiveness of nonpharmacological interventions in reducing fatigue in KTCs.

**Methods:**

Nonpharmacological interventions targeting fatigue in participants aged ≥18 years, who were either on the kidney transplantation waitlist or eligible candidates, were considered. A database search was conducted in PubMed, Embase, PsycINFO, CINAHL, and Web of Science. Results were reported in accordance with the guidelines provided by the Preferred Reporting Items for Systematic Reviews and Meta-analyses Protocols extension for Scoping Reviews Checklist.

**Results:**

In total, 67 studies were included. Interventions were divided into manipulative and body-based practices, exercise, mind-body therapies, energy healing, and combined interventions. Thirty-eight studies (76%) demonstrated a significant effect on fatigue, with effect sizes ranging from 0.43 to 4.85. Reflexology, massage therapy, progressive muscle relaxation, and acupressure combined with massage therapy showed the strongest significant intervention effects on fatigue and had the strongest study quality. However, the overall study quality was weak, particularly concerning confounding control, blinding procedures, and withdrawals and dropouts.

**Conclusions:**

Manipulative and body-based interventions showed the strongest significant effects on fatigue with the highest study quality. These interventions underscore the multifactorial nature of fatigue by targeting both its physical and psychological dimensions. Future high-quality research is needed to determine the optimal strategy for managing fatigue in KTCs.

## Teaser

Patients on the kidney transplant waiting-list may find relief from fatigue through various manipulative and body-based interventions, such as acupressure, massage, and muscle relaxation.

## Introduction

Kidney failure is the final stage of chronic kidney disease, requiring renal replacement therapy for survival.^1^ Kidney transplantation is the most desired treatment for patients with kidney failure, compared with dialysis.^2–4^ However, its availability is constrained by various factors, including a shortage of donor organs, which necessitates placing eligible patients on a kidney transplant waiting list. While the majority of kidney transplants are performed after the initiation of dialysis, a smaller subset of patients receives a transplant prior to the need for dialysis.^5^

During the waitlist period, fatigue is one of the most commonly reported symptoms among kidney transplant candidates (KTCs),^6–10^ affecting 60%-97% of this patient group.^9,11,12^ Fatigue is defined as a subjective overwhelming feeling of mental or physical exhaustion that impedes everyday functioning and quality of life.^13–15^ Fatigue severity in hemodialysis patients is reported as one of the highest among individuals with a chronic condition, including cancer patients undergoing chemotherapy treatment and patients with depression.^16^ Furthermore, higher fatigue levels are associated with a decrease in quality of life and daily activities, an increased risk for cardiovascular events, and higher mortality rates.^17–20^ A study by Bossola et al.^21^ showed that fatigued hemodialysis patients have a higher number of physical and emotional symptoms compared with nonfatigued patients, including restless legs, concentration problems, sexual dysfunction, and feeling sad.

The complexity of fatigue in chronic kidney disease lies in its multifaceted nature, involving physical, psychological, and emotional elements.^22^ While the precise causes of fatigue in KTCs remain largely unknown, multiple studies have shown that fatigue is related to comorbidity, poor physical functioning, and increased body mass index.^11,12,20,23–26^ Psychosocial factors that have been found to be associated with fatigue in KTCs include depression, anxiety, sleeping problems, lack of social support, and loneliness.^11,12,27–30^

Effective fatigue management can contribute in enhancing overall health in KTCs, influencing their physical and psychological well-being.^31^ Fatigue can be treated with pharmacological and nonpharmacological methods. Over the years, studies have explored various nonpharmacological interventions to reduce fatigue in KTCs, including exercise, yoga, relaxation, acupressure, acupuncture, aromatherapy, and massage therapy.^23,32–36^ Nonpharmacological interventions to reduce fatigue often make use of an holistic approach, addressing both physical and mental health, and thereby enhance the overall well-being. In addition, they may provide KTCs with a feeling of control and enhance their self-management.^37^ However, despite growing evidence, a comprehensive review on the effectiveness of nonpharmacological interventions in reducing fatigue in KTCs is yet to be conducted.

This scoping review explores the effectiveness of nonpharmacological interventions in reducing fatigue in KTCs. The objectives of this review were to summarize the effects of nonpharmacological interventions used to reduce fatigue and to examine challenges and limitations of different types of interventions targeting fatigue in KTCs.

## Methods

This scoping review was conducted in accordance with the Joanna Briggs Institute (JBI) methodology for scoping reviews^38^ and was guided by the Preferred Reporting Items for Systematic Reviews and Meta-analyses Protocols extension for Scoping Reviews (PRISMA-ScR) checklist.^39^ The methodological quality of eligible documents was assessed using the “Quality Assessment Tool for Quantitative Studies,” developed by the Effective Public Health Practice Project (EPHPP).^40^

### Protocol and registration

The protocol was registered prospectively with Figshare, a web-based interface designed for academic research data management and research data dissemination, on 2 June 2023.

### Eligibility criteria

Eligibility criteria were guided by the Participant, Concept, and Context Framework recommended by the JBI for scoping reviews.^38^

#### Participants

Adult (≥18 years) patients with chronic kidney disease, including kidney failure, both on dialysis and not on dialysis, waitlisted for kidney transplantation or who are potential candidates for kidney transplantation.

#### Concept

Any type of nonpharmacological intervention aimed at reducing fatigue in the study population. Nonpharmacological interventions are defined as interventions that do not involve the use of medications or pharmaceutical agents such as massage therapy, aromatherapy, or aerobic exercise. Studies were included if fatigue was reported as the primary outcome, one of the primary outcomes, or a secondary outcome.

#### Context

No limitations were set regarding the context of reviews. Studies from all countries and practice settings were included.

### Types of sources

All study designs that assessed the effects of a specific intervention on fatigue, including randomized controlled trials (RCTs), controlled clinical trials (CCTs)/quasi-experimental designs, cohort studies (1 or 2 group prepost designs), and interrupted time series were included.

### Search strategy

The search strategy involved 3 stages and was created in cooperation with a librarian. First, an initial search was conducted using the online databases PubMed, Embase, and Google Scholar. The initial search terms were formed using terms of the population, concept, and context of the study in the title. Synonyms for each term were also identified. In the second stage, the keywords in the title and abstract and the index terms of the retrieved articles were analyzed. All identified terms were used in a second search throughout the following databases on 3 January 2022: PubMed, Embase, PsycINFO, CINAHL, and Web of Science. Box 1 presents the search string utilized in PubMed. The search strategies for the remaining databases are outlined in Electronic Supplementary Material 1. An additional search was conducted in the same databases on December 15, 2023 to include newly published articles. Finally, the reference list of the identified articles for full text review was examined.

Box 1 **Search String PubMed**

(“Renal Insufficiency”[Mesh] OR “Renal Dialysis”[Mesh] OR chronic kidney disease[tiab] OR CKD[tiab] OR end stage renal disease[tiab] OR hemodialys*[tiab] OR haemodialys*[tiab] OR kidney failure[tiab] OR renal insufficiency[tiab] OR ((waitlist*[tiab] OR waiting list[tiab]) AND (renal[tiab] OR kidney[tiab])) OR dialys*[tiab] OR kidney transplant candidates[tiab] OR renal failure[tiab] OR ((pre emptive[tiab] OR preemptive) AND (renal[tiab] OR kidney[tiab])) OR end stage kidney disease[tiab]) AND (“Fatigue”[Mesh] OR fatigue[tiab] OR tired*[tiab] OR exhaustion[tiab] OR fatigability[tiab]) AND (“Psychosocial intervention”[Mesh] OR “Sleep Hygiene”[Mesh] OR intervention*[tiab] OR program*[tiab] OR training[tiab] OR counsel*[tiab] OR psychotherap*[tiab] OR sleep hygiene[tiab])

The searches were conducted without any language and publication date limitations. However, only studies published in the English or Dutch language were considered for inclusion. In total, four authors were contacted for additional information on three dates (September 21, 2022, October 12, 2022, and January 31, 2024). Two provided additional details, while the other 2 did not share further information regarding their study population.^41,42^

### Selection of sources of evidence

The identified documents were imported into CADIMA, a web tool that facilitates the process and assurance of the documentation of reviews. After the removal of duplicates, 2 independent reviewers (H.M. and A.H. or C.A. and A.H.) screened the titles and abstracts identified by the literature search based on the review inclusion criteria. To determine interrater reliability, a calibration exercise was performed by pilot testing a random sample of 100 citations. A kappa of 0.72 was achieved. Next, 2 reviewers screened the remaining titles and abstracts. Subsequently, they reviewed the full-text articles to determine their conformity with the eligibility criteria. Discrepancies were resolved by discussion and obtaining consensus between two reviewers and by a third independent reviewer (C.A. or H.M.).

During the process, we decided to exclude one study due to insufficient information, as it did not report any results.^43^ Additionally, 4 studies with a sample size of 10 or less were excluded due to potential limitations in generalizability of their findings.^44–47^ In cases (*n* = 3) where multiple articles covered the same study and intervention,^48,49^ we selected the article with the largest study population for inclusion.^50^

### Data charting

A data charting form in Microsoft Excel was developed and discussed with 3 reviewers (A.H., H.M., and C.A.) to determine which variables to extract from the included documents. Reviewer A.H. extracted the data, charted the data, discussed the results with reviewers H.M. and C.A., and continuously updated the data-charting form to maximize clarity and accuracy. The following data items were extracted from each included document: first author, year of publication, country of origin, study design, population, sample size, demographical data, intervention information (type, duration, setting, and measurement points), and validated outcome measures to assess fatigue. Intervention type was divided into manipulative and body-based practices, exercise, mind-body therapies, energy healing, or combined interventions.

### Critical appraisal of individual sources of evidence

The quality of the studies included was assessed using the 8 domains from the EPHPP: selection bias, study design, confounders, blinding, data collection method, withdrawals/dropouts, intervention integrity, and analyses. This tool, together with a user manual, provides a standardized means to assess the research quality and develop recommendations for study findings. According to the guidelines, each domain is categorized as strong, moderate, or weak. A study is considered strong if there are no weak ratings across the first 6 domains, indicating a high level of methodological rigor, with minimal bias and high reliability and validity of the results. A study is considered moderate if there is 1 weak rating, reflecting some methodological limitations; however, the findings are generally considered reliable with a moderate risk of bias. A study is considered weak if there are 2 or more weak ratings, indicating considerable methodological limitations or biases that may compromise the reliability or validity of the study findings.

The quality of the studies was evaluated by 2 reviewers (H.M. and A.H. or C.A. and A.H.), with discrepancies resolved by discussion and obtaining consensus between two reviewers and by a third independent reviewer (C.A. or H.M.).

### Synthesis of results

The collected data were collated and analyzed, and results and outcomes were reported in a descriptive numerical summary and tabulation of findings. Additionally, the findings were presented in a narrative synthesis, grouped by the studies’ general characteristics, types of interventions, and quality and effectiveness. The interventions were categorized according to the classification system of the US National Center for Complementary and Alternative Medicine,^51,52^ which distinguishes between various types of complementary and alternative medicine interventions. The categorization process involved a detailed analysis of the intervention’s content and delivery method and was thoroughly discussed and agreed upon by all authors. Additionally, the potential implications that findings hold for future research were discussed. When sufficient data were available, the effect size (ES) within and between groups was estimated according to Cohen’s *d* criteria. Alternatively, if data were insufficient, the *P*-value was documented.^53^ A positive ES indicates an improvement and a negative indicates deterioration in the group, with ES ≥ 0.2 considered weak, ES ≥ 0.5 considered moderate, and ES ≥ 0.8 considered strong. The ES was considered significant when its 95% CI excluded the null value. No formal power analysis was performed; however, a priori and post hoc power analyses were reported when documented in the studies.

## Results

### Selection of sources of evidence

**Figure 1.**
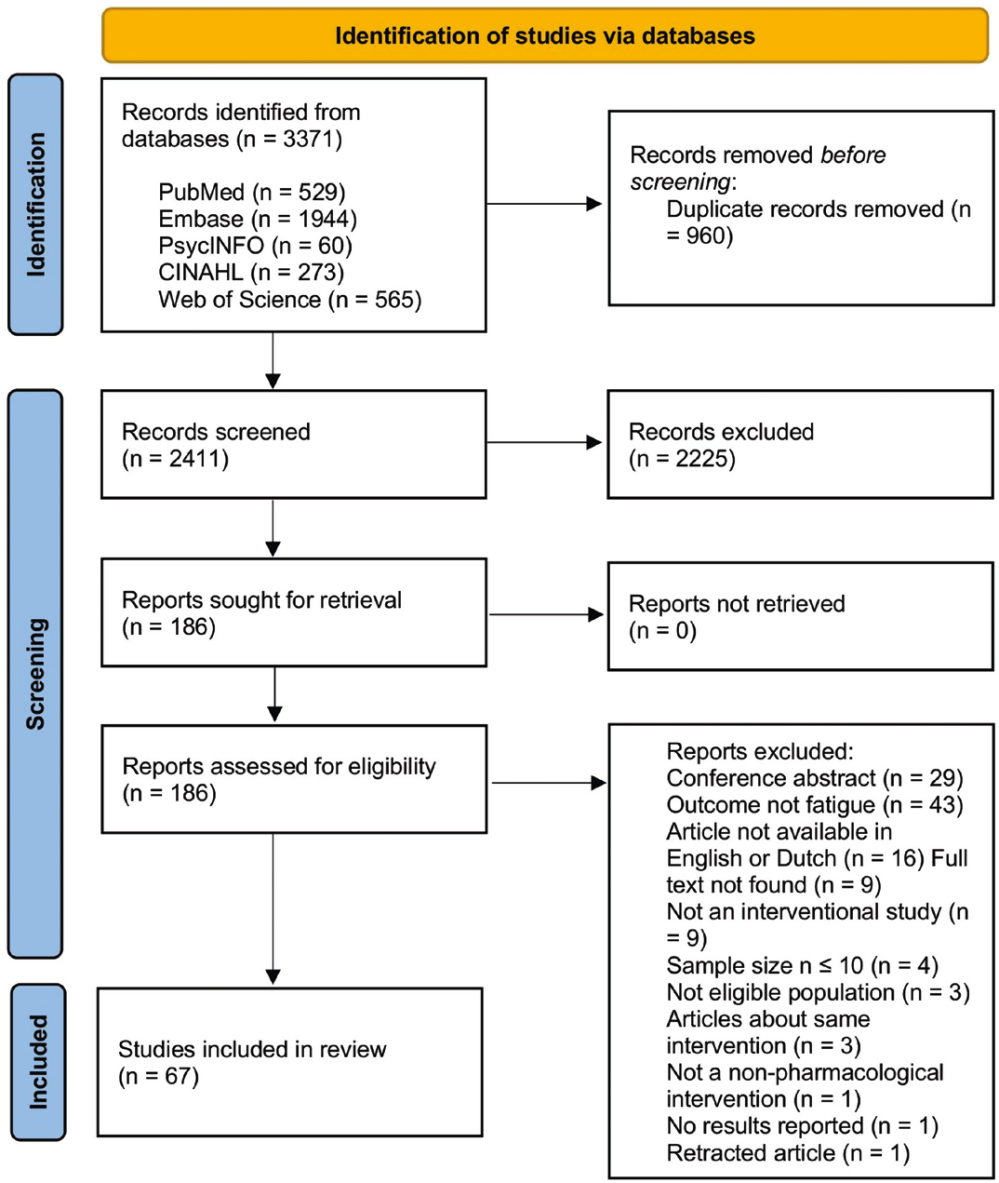
Scoping review flow chart.

The database search identified 3371 potentially relevant documents (PubMed [*n* = 529]; Embase [*n* = 1944]; PsycINFO [*n* = 60]; CINAHL [*n* = 273]; and Web of Science [*n* = 565]). Following duplicate removal, 2411 articles were screened on title and abstract, resulting in 186 potentially eligible full-text articles based on study population, outcome measure, study design, and type of intervention. A further 119 were excluded based on review of the full text. A total of 67 were included in this review. The outcomes of the study selection process are presented in a flow chart (Figure 1).

### Methodology and setting of included studies

The included studies were published between 1999 and 2023. Five different study designs were employed across the studies, with the majority utilizing a RCT (*n* = 39; 57%) or CCT design (*n* = 20; 30%). Most of these studies utilized a 2-arm design (*n* = 51; 86%), while 7 studies (12%) employed a 3-arm design and one study utilized a 4-arm design (1%). The remaining studies included cohort studies (1 group pre + post, *n* = 6; 9%), analytic cohort studies (2 groups pre + post, *n* = 1; 2%), and an interrupted time series design (*n* = 1; 2%). Out of the 67 studies, 40 (60%) reported a power analysis.

The study population exhibited a high level of homogeneity, with nearly all studies encompassing hemodialysis patients (*n* = 63; 94%). Other study populations included peritoneal dialysis patients (*n* = 1; 2%), chronic kidney disease patients with and without dialysis (*n* = 1; 2%), and nondialysis patients (*n* = 1; 2%). The mean sample size was 70.3 (±41.1), with a median of 61 and a mode of 60. Sample sizes varied widely, ranging from 13 to 220 participants. The majority of the studies were conducted in the dialysis unit (*n* = 51; 77%), while other settings included the combination of dialysis unit and home-based (*n* = 8; 12%), entirely home-based (*n* = 5; 8%), or a hospital setting (*n* = 2; 3%). The studies were conducted in 16 different countries, with the majority of the included studies originating from countries in Asia (*n* = 57; 85%), with Iran (*n* = 18; 32%) and Turkey (*n* = 14; 25%) as primary contributors.

Fatigue was the primary outcome of 26 studies, 1 of the primary outcomes in 34 studies, and a secondary outcome in 7 studies. Eight studies (12%) employed multiple outcome measures of fatigue. Furthermore, across the included studies, 18 outcome measures for fatigue were utilized. Seventeen of these were shown to be valid and reliable, as reported in the included studies or separate publications. The fatigue outcome measures were assessed for content validity and internal consistency, aligning with the EPHPP data collection method criteria. However, we were unable to verify the content, validity, and reliability of the outcome measure Fatigue scale by Chen and Ku (1998) utilized in one study.^54^ The fatigue questionnaires varied in length and scale type, ranging from a single item on a 5-point Likert scale (Postdialysis Fatigue) to 22 items scored on a 0-10-point scale (Piper Fatigue Scale [PFS]). Commonly used self-reported outcome measures were the Fatigue Severity Scale (*n* = 17; 25%) and the PFS (*n* = 15; 22%).

A detailed summary of all included studies and an overview of the quality assessment are available in Electronic Supplementary Material 2 and 3.

### Intervention types

The interventions used in the included studies were divided into 5 categories: manipulative and body-based practices, exercise, mind-body therapies, energy healing, and combination of interventions. An explanation of these categories is presented in Box 2.

Box 2 **Explanatory Intervention Categories**

**Manipulative and Body-Based Practices** Involves a practitioner using their hands or techniques to directly address the structure of the body, with a primarily focus on physical structures and systems of the body, including bones, joints, soft tissues, and occasionally the circulatory and lymphatic systems.^98^

**Exercise** Is planned, structured, and repetitive, with a focus on improving or maintaining physical fitness through aerobic, resistance, balance and/or flexibility exercises.^99^

**Mind-Body Therapies** Addresses the interaction between the body, mind, brain, and behavior through a combination of mental focus, breathing, and body movements. Its purpose is to use the mind to influence physical function and enhance overall health.^100^

**Energy Healing** Is a practice based on a belief that a vital energy flows through the body. Techniques focus on manipulating the energy field, with the goal of restoring balance and improving the energy flow.^101^

**Combination of interventions** Comprised of two or more interventions from different intervention categories.

Twenty studies (30%) addressed the effect of manipulative and body-based practices on fatigue, followed by exercise (*n* = 16; 24%), combined interventions (*n* = 14; 21%), mind-body therapies (*n* = 13; 19%), and energy healing (*n* = 4; 6%). The interventions varied widely across the studies, with a combination of resistance and aerobic exercises (*n* = 5; 7%) and acupressure (*n* = 5; 7%) emerging as the most prevalent interventions. In over three-quarters of the studies (*n* = 52), the intervention effect was assessed immediately after the intervention. In the remaining studies (*n* = 15), this assessment ranged from 1 week to 6 months postintervention. Table 1 outlines an overview of all interventions.

**Table 1:** | Overview of types of interventions.

Intervention categories	Interventions	Number of studies
Manipulative and body-based practices	Acupressure	5
	Massage therapy	4
	Progressive muscle relaxation	3
	Reflexology	2
	Footbath	2
	Herbal point therapy	1
	Thermal gel compresses (3 groups)	1
	Acupressure and massage therapy	1
	Massage therapy and reflexology (2 groups)	1
Exercise	Combination of resistance and aerobic exercises	5
	Aerobic exercise	4
	Combination of resistance and flexibility exercises	2
	Resistance exercise	2
	Combination of resistance, aerobic, and flexibility exercises	1
	Combination of resistance, flexibility, and balance exercises	1
	Kinesitherapy with ELF-PEMF	1
Mind—body therapies	Aromatherapy	4
	Music therapy	3
	Mindfulness	1
	Cognitive behavioral therapy	1
	Qigong	1
	Pranayama	1
	Pranayama and breathing exercises (3 groups)	1
	Sensory stimulation	1
Energy healing	Reiki	2
	Energy management	1
	Sleep hygiene education	1
Combined interventions	Cognitive behavioral therapy and sleep hygiene education	2
	Exercise education, and resistance, and aerobic exercise	1
	Health education and aerobic exercise	1
	Health education and exercise	1
	Health education and cognitive behavioral therapy (2 groups)	1
	Progressive muscle relaxation and aerobic exercise (3 groups)	1
	Benson muscle relaxation and aromatherapy (3 groups)	1
	Benson muscle relaxation and music therapy	1
	Active range of motion (AROM) and deep breathing exercise	1
	AROM and yoga	1
	Resistance and breathing exercises	1
	Aromatherapy and aromatherapy with massage therapy (3 groups)	1
	Massage therapy and audio recording (religious)	1

### Findings per intervention type

#### Manipulative and body-based practices

A total of 20 studies were included, in which 9 interventions were explored. Five of these interventions were commonly applied across the studies: acupressure, progressive muscle relaxation, massage therapy, reflexology, and footbath. Acupressure (*n* = 5; 25%) and massage therapy (*n* = 4; 20%) were the most prevalent components of the interventions. Intervention durations ranged from 1 day to 12 weeks, with frequencies varying from a single session to daily sessions. Session length ranged from 5 minutes to 4 hours. The sample size had a mean of 83.4 (±38.5), with a median of 74.5. The range of sample sizes varied from 27 to 200.

##### Effectiveness

Effect sizes were calculated in fourteen of the 20 studies (70%), with 13 studies (93%) presenting a significant intervention effect, ranging between 0.54 and 3.06.^41,42,55–65^ The studies with the largest intervention effect on fatigue included thermal gel compresses (ES = 3.06),^55^ footbath (ES = 2.72),^65^ and acupressure (ES = 1.95).^64^ Out of the 13 studies, 7 (54%) documented a power analysis (54%), achieving a sample size that resulted in a power (1 − β) of ≥0.80.^56–59,65^ In almost all studies (*n* = 17; 85%), the intervention effect was assessed immediately after the intervention.

##### Quality assessment

In 10 of the studies (45%), the quality was considered weak; 7 studies (35%) were rated as moderate and 4 (20%) as strong. Concerns were identified regarding selection bias, blinding procedures, and withdrawals and dropouts in most of the studies. In half of the studies (*n* = 10), the percentage of selected participants that agreed to participate was unclear, indicating concerns regarding participant selection. In almost half of the studies, either the outcome assessors and/or participants were not blinded, or the blinding process was unclear. In 8 studies (40%), both the outcome assessor and the participants were not blinded. In a quarter of the studies (*n* = 5), the withdrawal and dropouts were unclear.

#### Exercise

In the 16 studies, 7 types of exercise interventions were used, of which 4 were commonly employed: aerobic exercise, resistance exercise, a combination of resistance and aerobic exercises, and a combination of resistance and flexibility exercises. The most frequently used intervention was a combination of aerobic and resistance exercises (*n* = 5; 31%). Overall, 4 studies (25%) employed a combination of different exercise intervention types, while 12 studies (75%) used a single type of exercise intervention. Intervention durations varied from 2 to 36 weeks, except for kinesitherapy, which lasted 3 years with 40 sessions annually. Intervention frequencies ranged from twice daily to 3 times a week, while session durations ranged from 8 to 80 minutes. The sample size had a mean of 48.3 (±25.9), with a median of 41.5. The range of sample sizes varied from 20 to 124.

##### Effectiveness

Effect sizes were calculated in 13 of the 16 studies (81%), with 8 studies (62%) demonstrating a significant intervention effect on fatigue, ranging between 0.68 and 3.86.^66–73^ The studies with the largest intervention effect on fatigue included resistance exercises (ES = 3.86 and 1.89),^66,70^ kinesitherapy (ES = 1.90),^67^ and a combination of resistance with balance and flexibility or aerobic exercises (ES = 1.87 and 1.79).^68,69^ Half of these studies (*n* = 4) documented a priori power analysis, with 3 studies achieving a sample size resulting in a power (1 − β) of ≥0.80.^68,69,73^ In more than 80% of the studies (*n* = 13), the intervention effect was measured immediately after the intervention.

##### Quality assessment

Seven of the 16 studies (44%) were classified as having weak quality, 7 (44%) as moderate, and 2 (12%) as strong. There were concerns regarding selection bias, confounding control, blinding procedures, and withdrawals and dropouts in some studies. Twelve studies received a moderate rating and 1 study received a weak rating, indicating potential selection bias. In half of the studies (*n* = 8), it remained unclear whether there were significant differences between groups prior to the intervention, indicating potential confounding factors. In 94% of the studies (*n* = 15), either the outcome assessors and/or the participants were not blinded, or the blinding process was unclear. In 3 studies (19%), withdrawals and dropout rates were either >60% or unclear.

#### Mind-body therapies

Of the 13 studies, 8 interventions were employed, with aromatherapy (*n* = 4; 31%) and music therapy (*n* = 3; 23%) most commonly applied across studies. Intervention durations varied from 1 day to 24 weeks, with frequencies ranging from a single session to daily sessions. Session durations ranged from 2 minutes to 3 hours. The sample size had a mean of 63.2 (±35.7), with a median of 60. The range of sample sizes varied from 24 to 172.

##### Effectiveness

Effect sizes were calculated in all 13 studies, of which 9 (69%) presented a significant intervention effect, with ESs ranging between 0.70 and 4.85.^74–82^ The studies with the largest intervention effect on fatigue included Pranayama (ES = 4.85),^82^ music therapy (ES = 3.56),^81^ and aromatherapy (ES = 3.05).^80^ Of the 9 studies, 6 documented a power analysis, with 4 studies achieving a sample size resulting in a power (1 − β) of ≥0.80.^74–76,80^ In 10 studies (77%), the intervention effect was measured immediately postintervention.

##### Quality assessment

The study quality was considered weak in 7 of the 13 studies (54%), moderate in 5 studies (38%), and strong in 1 study (8%). There were concerns regarding selection bias, blinding procedures, and withdrawals and dropout throughout studies. Information regarding the participation rate was not provided in 6 studies (46%), and participation rate was less than 60% in 3 studies (23%). In 11 studies (85%), the blinding process was either not described or the outcome assessor and/or the participants were not blinded. In only 1 study, both the outcome assessor and the participants were blinded. Regarding withdrawals and dropouts, 4 studies were rated weak (31%). In 3 of these studies, the percentage of participants completing the study was not described, and in 1 study, the participation rate of completing the study was less than 60%.

#### Energy healing

In the 4 studies, 3 different interventions were employed, including Reiki, energy management, and sleep hygiene education. Among these, Reiki was the most commonly addressed (*n* = 2; 50%). The intervention period in these studies ranged from 1 day to 7-9 weeks, with frequencies varying from once daily to 3 times a week. The duration of a session ranged from 30 to 60 minutes. The sample size had a mean of 55.8 (±21.1), with a median of 60.5. The range of sample sizes varied from 22 to 80.

##### Effectiveness

Three of the 4 studies presented a significant intervention effect on fatigue, ranging between 0.43 and 2.12. These interventions included Reiki (ES = 2.12 and 0.95)^83,84^ and sleep hygiene education (ES = 0.43).^85^ All 3 studies documented a priori power analysis, resulting in a power (1 − β) of ≥0.80. The assessment of the intervention effect ranged from immediately after the intervention to 12 weeks postintervention.

##### Quality assessment

The study quality was considered weak in all 4 studies, with concerns regarding selection bias, confounding control, blinding procedures, and withdrawals and dropouts. In half of the studies, either the percentage of individuals who agreed to participate was unclear or it was less than 60%, indicating concerns regarding participant selection. In the majority of the studies, the percentage of confounders controlled for was either unclear (*n* = 2; 50%) or less than 60% (*n* = 1; 25%). The blinding process was described in 3 studies (75%) with none of the outcome assessors blinded and 1 study blinding the study participants (25%). Next to this, in half of the studies, the percentage of participants completing the study was unclear or less than 60%.

#### Combination of interventions

In total, across the 14 studies, 13 different combinations of intervention types were considered, such as manipulative and body-based practices with mind-body therapies (*n* = 4), exercise with mind-body therapies (*n* = 3), and exercise with education (*n* = 3). The intervention periods in these studies ranged from 3 to 24 weeks, with frequencies ranging from once daily to 3-5 times a week. The duration of a session ranged from 15 to 90 minutes. The sample sizes had a mean of 87.4 (±52.7), with a median of 88.5 and a range between 13 and 220.

##### Effectiveness

Effect sizes were calculated in 7 of the 14 studies (50%), with 5 studies (71%) showing a significant intervention effect on fatigue, ranging between 0.56 and 3.01.^86–90^ The studies with the largest intervention effect on fatigue included massage therapy with listening to religious audio recording (ES = 3.01),^90^ and Benson muscle relaxation and aromatherapy (2.96).^89^ Four out of the 5 studies (80%) documented a priori power analysis, all of which achieved a sample size resulting in a power (1 − β) of ≥0.80.^87–90^ In 11 of the 14 studies, the effect was assessed immediately postintervention (78%).

##### Quality assessment

The study quality was categorized as weak (*n* = 4; 29%) or moderate (*n* = 10; 71%) and concerns were identified regarding selection bias and blinding procedures. In most studies, the participation rate was either not described (*n* = 6; 43%) or less than 60% (*n* = 2; 14%). Only 3 studies (21%) blinded the outcome assessor(s). In the remaining studies (*n* = 11; 79%), neither the outcome assessors and/nor participants were blinded, or the blinding process was unclear.

## Discussion

### Summary of evidence

In this scoping review, we identified 67 studies addressing nonpharmacological interventions targeting fatigue in KTCs across various settings. Among the 40 different interventions utilized, a combination of resistance and aerobic exercises, and acupressure were the most prevalent. This highlights the growing recognition of both exercise and manual therapies in addressing fatigue. The interventions with the largest significant effect on reducing fatigue in KTCs and the highest study quality included reflexology, massage therapy, progressive muscle relaxation, and acupressure combined with massage therapy. These interventions were all categorized in the manipulative and body-based practice category, which focus on physical techniques that influence the body’s structure and function. Reflexology targets both physical and psychological well-being, while progressive muscle relaxation, as well as acupressure combined with massage therapy, aim to alleviate physical symptoms of fatigue and reduce stress. These findings suggest that interventions addressing both the physical and psychosocial dimensions of fatigue are particularly effective, emphasizing the multifactorial nature of fatigue and the need for comprehensive, integrative approaches to its management.

However, the studies included in this review demonstrated considerable diversity in methodology, quality, duration, sample size, and fatigue outcome measures. Additionally, nearly all studies focused on hemodialysis patients, with most interventions conducted during dialysis in a dialysis center. Notably, half of the studies with a significant intervention effect on fatigue did not report a power analysis. Furthermore, the most common intervention showing a significant ES was aromatherapy (*n* = 4; 14%), although 2 studies did not achieve a sample size resulting in a self-determined power (1 − β) of 0.90. Despite generally strong ratings for study design and data collection methods, concerns were raised regarding selection bias, blinding procedures, confounding control, and withdrawals and dropout rates in the majority of studies. The studies included often lacked information on these aspects, potentially leading to an overestimation of study quality. Conversely, it should be acknowledged that blinding participants and/or outcome assessors is not always feasible for certain interventions.

Furthermore, the homogeneity of the study population, consisting primarily of hemodialysis patients, may limit the generalizability of the results to nondialysis KTCs. Additionally, the majority of the intervention effects were assessed immediately postintervention, limiting the understanding of their long-term effects. Moreover, over 3-quarters of the studies were conducted in Asian countries, potentially influencing the generalizability of the interventions to other regions due to cultural differences.

### Strengths and limitations

The strength of this review lies in its broad search criteria, which did not limit the inclusion of studies based on specific study designs, such as RCTs, modes of delivery, such as home-based interventions, or specific intervention types, such as physical activity. However, our review does have limitations. First, we were unable to calculate ESs in 17 (25%) of the studies included, restricting the ability to quantify and compare the magnitude of effects across all studies, impacting the overall reliability and interpretability of the review’s findings. Second, caution is necessary when interpreting the calculated ESs, as they were derived from various fatigue outcome measures, generally low sample sizes, and various study designs. This may lead to inconsistencies in the findings, including potential overestimation of the intervention effect and the introduction of biases such as selection, attrition, and detection bias.

## Conclusions

The aim of this scoping review was to explore the effectiveness of nonpharmacological interventions in reducing fatigue in KTCs. Several promising interventions were identified, including reflexology, massage therapy, progressive muscle relaxation, and acupressure combined with massage therapy. These interventions underscore the multifactorial nature of fatigue as they target both the physical and psychological dimensions of fatigue. This highlights the necessity for comprehensive, integrative strategies in the management of fatigue in KTCs.

**Implication for Research and Practice**

The variety of effective interventions identified in this review provides the opportunity to offer a range of strategies for managing fatigue in KTCs. A personalized approach tailored to patients’ preferences allows for flexibility in selecting interventions that best suit individual needs and circumstances.

Among the identified interventions, progressive muscle relaxation can be implemented relatively easy in practice settings. KTCs can independently practice progressive muscle relaxation after receiving training and instruction, requiring minimal supervision, and involving low material costs.^61^ However, progressive muscle relaxation necessitates regular practice and relies on patient motivation.^91^ Regular monitoring can enhance motivation and help address any challenges KTCs may encounter,^61^ which is consistent with findings reported in cancer patients.^92^ In contrast, interventions such as reflexology, massage therapy, and acupressure may be more difficult to implement in clinical practice as this requires the deployment of skilled healthcare professionals, which can increase costs. These interventions are typically performed by professionals, which could affect their feasibility and sustainability in healthcare settings. Training healthcare providers, such as nurses, to deliver these interventions has shown to increase the availability of skilled practitioners.^93,94^ Additionally, digital platforms offering guided instructions can enable KTCs to practice these interventions at home, enhancing their feasibility and sustainability.^95^

Future research should involve larger, more geographically diverse studies with long-term follow-up and consistent fatigue outcome measures. The use of Patient-Reported Outcome Measures can enhance the comparability of intervention effects on fatigue in KTCs.^96,97^ Furthermore, future research should focus on interventions with strong evidence for efficacy and safety in managing fatigue in KTCs while also considering their ease of implementation in clinical practice. There is a lack of research addressing the long-term effectiveness of these interventions and their practical feasibility, including cost-effectiveness and adaptability to routine clinical practice. Additionally, improving the rigor of the methods used is essential, such as addressing bias, controlling confounders, and utilizing blinding procedures where applicable.

## Supplementary Material

kaaf017_suppl_Supplementary_Materials

## Data Availability

De-identified data from this study are not available in an a public archive. De-identified data from this study will be made available (as allowable according to institutional IRB standards) by emailing the corresponding author.

## Open Science Transparency Statements

(1)Study registration: The study was preregistered at Figshare (https://doi.org/10.6084/m9.figshare.19958873.v1).

(2)Analytic plan preregistration: The analysis plan was not formally preregistered.

(3)Analytic code availability: Study quality was assessed using the “Quality Assessment Tool for Quantitative Studies,” developed by the Effective Public Health Practice Project (EPHPP). Effect sizes within and between groups were calculated using the URL: https://www.psychometrica.de/effect_size.html.

(4)Materials availability: The included studies in this review are publicly available via PubMed.
