# Supplementary material for: Insights into effective fatigue reducing interventions in kidney transplant candidates: a scoping review
Source: Ann Behav Med. 2025 Mar 14;59(1):kaaf017. doi: 10.1093/abm/kaaf017 (PMC11907435; doi:10.1093/abm/kaaf017)
Supplement: kaaf017_suppl_Supplementary_Materials [file kaaf017_suppl_supplementary_materials.docx]

# **Electronic Supplementary Material 1**

# *Search Strategies*

## **Embase**

('kidney failure'/exp OR 'dialysis'/exp OR 'chronic kidney disease':ab,ti OR ckd:ab,ti OR 'end stage renal disease':ab,ti OR hemodialys*:ab,ti OR haemodialys*:ab,ti OR 'kidney failure':ab,ti OR 'renal insufficiency':ab,ti OR dialys*:ab,ti OR 'kidney transplant candidates':ab,ti OR 'renal failure':ab,ti OR 'end stage kidney disease':ab,ti OR ((renal:ab,ti OR kidney:ab,ti) AND (waitlist*:ab,ti OR 'waiting list':ab,ti)) OR ((renal:ab,ti OR kidney:ab,ti) AND ('pre emptive':ab,ti OR preemptive:ti,ab)))

AND ('fatigue'/exp OR fatigue:ab,ti OR tired*:ab,ti OR exhaustion:ab,ti OR fatigability:ab,ti)

AND ('psychosocial intervention'/exp OR intervention*:ab,ti OR program*:ab,ti OR training:ab,ti OR counsel*:ab,ti OR psychotherap*:ab,ti OR 'sleep hygiene':ab,ti) AND [embase]r/lim

## **PsycINFO**

(DE "Kidney Diseases" OR DE "Dialysis" OR DE "Hemodialysis" OR TI (“chronic kidney disease” OR CKD OR “end stage renal disease” OR hemodialys* OR haemodialys* OR “kidney failure” OR “renal insufficiency” OR ((waitlist* OR “waiting list”) AND (renal OR kidney)) OR dialys* OR “kidney transplant candidates” OR “renal failure” OR ((“pre emptive” OR preemptive) AND (renal OR kidney)) OR “end stage kidney disease”) OR AB (“chronic kidney disease” OR CKD OR “end stage renal disease” OR hemodialys* OR haemodialys* OR “kidney failure” OR “renal insufficiency” OR ((waitlist* OR “waiting list”) AND (renal OR kidney)) OR dialys* OR “kidney transplant candidates” OR “renal failure” OR ((“pre emptive” OR preemptive) AND (renal OR kidney)) OR “end stage kidney disease”))

AND (DE "Fatigue" OR TI (fatigue OR tired* OR exhaustion OR fatigability) OR AB (fatigue OR tired* OR exhaustion OR fatigability))

AND (DE "Psychosocial Rehabilitation" OR DE "Intervention" OR DE "Treatment" OR DE "Early Intervention" OR DE "Family Intervention" OR DE "Group Intervention" OR DE "Prevention" OR DE "Rehabilitation" OR TI (intervention* OR program* OR training OR counsel* OR psychotherap* OR Sleep Hygiene) OR AB (intervention* OR program* OR training OR counsel* OR psychotherap* OR Sleep Hygiene))

## **CINAHL**

((MH "Renal Insufficiency+") OR (MH "Dialysis+") OR TI (“chronic kidney disease” OR CKD OR “end stage renal disease” OR hemodialys* OR haemodialys* OR “kidney failure” OR “renal insufficiency” OR ((waitlist* OR “waiting list”) AND (renal OR kidney)) OR dialys* OR “kidney transplant candidates” OR “renal failure” OR ((“pre emptive” OR preemptive) AND (renal OR kidney)) OR “end stage kidney disease”) OR AB (“chronic kidney disease” OR CKD OR “end stage renal disease” OR hemodialys* OR haemodialys* OR “kidney failure” OR “renal insufficiency” OR ((waitlist* OR “waiting list”) AND (renal OR kidney)) OR dialys* OR “kidney transplant candidates” OR “renal failure” OR ((“pre emptive” OR preemptive) AND (renal OR kidney)) OR “end stage kidney disease”))

AND (MH "Fatigue+" OR TI (fatigue OR tired* OR exhaustion OR fatigability) OR AB (fatigue OR tired* OR exhaustion OR fatigability))

AND (MH "Psychosocial Intervention" OR MH "Intervention Trials" OR TI (intervention* OR program* OR training OR counsel* OR psychotherap* OR “sleep hygiene”) OR AB (intervention* OR program* OR training OR counsel* OR psychotherap* OR “sleep hygiene”))

## **Web of Science**

TS=("renal insufficiency" OR "renal dialysis" OR "chronic kidney disease" OR CKD OR "end stage renal disease" OR hemodialys* OR haemodialys* OR "kidney failure" OR "renal insufficiency" OR ((waitlist* OR "waiting list") AND (renal OR kidney)) OR dialys* OR "kidney transplant candidates" OR "renal failure" OR (("pre emptive" OR preemptive) AND (renal OR kidney)) OR "end stage kidney disease")

AND TS=(fatigue OR tired* OR exhaustion OR fatigability)

AND TS=(“psychosocial intervention” OR “sleep hygiene” OR intervention* OR program* OR training OR counsel* OR psychotherap*)

# **Electronic Supplementary Material 2**

# *General Characteristics Included Studies*

| **First Author, Year, Country of Origin** | **Study Design and**  **Sample Characteristics Total N, N per Group, % Male, Age (years) Mean (SD)** | **Intervention(s) and Measurement Points** | **Outcome Measure Fatigue** | **Effectiveness Outcomes Fatigue ↑ = Sig. Increase  ↓ = Sig. Decrease  ≈ No Difference** | **Power Analysis**  **Sample Size** | **Quality Assessment** |
| --- | --- | --- | --- | --- | --- | --- |
| **Manipulative and Body-based Practice Interventions** | | | | | | |
| Ahmadidarrehsima, 2018, Iran [1] | CCT  HD patients (dialysis: ≥ 6 months; 3x/week)  N = 52  - I1 n = 26  57.7% male  Age NR  - I2 n = 26  80.8% male  Age NR | I1: 3 weeks, during dialysis, foot reflexology (30 min (15 min per foot), 2x/week, 5 sessions total) I2: 3 weeks, during dialysis, slow back stroke (10 min, 2x/week, 5 sessions total)  Assessments: - Pre-intervention - Post-intervention | FSS | ↓ within I1 d = -1.70, 95% CI [-2.596, -0.802]  ↓ within I2 d = -1.33, 95% CI [-2.177, -0.479] I1 ≈ I2 d = 0.29, 95% CI [-0.479, 1.067] | NR | Moderate |
| Biçer, 2021, Turkey [2] | RCT  HD patients (dialysis ≥ 6 months)  N = 135  - I n = 67  35.8% male  Age 64.0 (±11.6)  - C n = 68  44.1% male  Age 65.8 (±12.1) | I: 4 weeks, during dialysis, acupressure involving a electrostimulation device (60 min, 3x/week) C: 4 weeks, during dialysis, sham acupressure (60 min, 3x/week)  Assessments PFS: - Pre-intervention - Post-intervention  Assessments VAS: - Each week: after 1st, 2nd, 3rd session, FU at home | PFS  VAS-F | PFS ≈ within I d = -0.32, 95% CI [-0.799, 0.165] ≈ within C d = 0.27, 95% CI [-0.213, 0.742] I ≈ C d = -0.26, 95% CI [-0.599, 0.078]  VAS-F ↓ within I p < 0.001 at week 1 - 4 ↓ within C p < 0.001 at week 1 and week 4 I ↓ C p < 0.001 at week 4 (gradually increase in difference I - C from week 1 - 4) | Post hoc 0.89 | Weak |
| Cecen, 2021, Turkey [3] | RCT  HD patients (dialysis ≥ 6 months)  N = 82  - I1 n = 27  63% male  Age 53.1 (±18.1)  - I2 n = 27  33.3% male  Age 60.0 (±16.5)  - C n = 28  46% male  Age 55.4 (±1.0) | I1: 4 weeks, during dialysis, hand massage, non-fistula hand (8 min, 3x/week) I2: 4 weeks, during dialysis, foot massage (8 min (4 min per foot), 3x/week) C: Usual care  Assessments: - Baseline - 2 weeks after baseline - Post-intervention | VAS-F | Fatigue subdimension  ↓ within I1 p < 0.001 at 4 weeks after baseline  ↓ within I2 p < 0.001 at 4 weeks after baseline  ≈ within C p = 0.45 at 4 weeks after baseline  Energy subdimension ≈ within I1 p = 0.39 at 4 weeks after baseline ↓ within I2 p < 0.001 at 4 weeks after baseline ≈ within C p = 0.43 at 4 weeks after baseline | Unclear, sample size achieved | Moderate |
| Cho, 2004, Taiwan [4] | CCT  HD patients (dialysis ≥ 3 months)  N = 58  - I n = 28  28.6% male  Age 45.1 (±9.7)  - C n = 30  56.7% make  Age 53.7 (±8.5) | I: 4 weeks, during dialysis, acupressure (four acupoints (ST36, SP6, KI3, KI1); 3 min per point) and massage therapy (lower limb massage,15 min, 3x/week), performed by a researcher  C: Usual care  Assessments: - Pre-intervention - Post-intervention | PFS | ↓ within I d = -1.10, 95% CI [-1.658, -0.534] ≈ within C d = -0.12, 95% CI [-0.628, 0.385] I ↓ C d = -0.81, 95% CI [-1.345, -0.273] | A priori 0.8, sample size n = 26 per group | Moderate |
| Eglence, 2013, Turkey [5] | CCT  HD patients (dialysis≥ 3 months)  N = 118  - I n = 52  55.8% male  Age 52.4 (±11.6)  - C n = 66  48.5% male  Age 55.6 (±9.8) | I: 4 weeks, during dialysis, acupressure on four acupressure points (ST36, GB34, SP6, K1) using a transcutaneous electrical nerve stimulation acupuncture pen, by a trained investigator and two students (12 min, 3x/week) C: Usual care  Assessments: - Pre-intervention - 1 month post-intervention | VAS-F  PFS | VAS ↓ within I p < 0.001 ≈ within C p = 0.069 I ↓ C p < 0.001  PFS ↓ within I p < 0.001 ≈ within C p = 0.178 I ↓ C p = 0.002 | A priori 0.85, sample size n = 118 in total | Moderate |
| Ghozhdi, 2023, Iran [6] | RCT  HD patients (dialysis ≥ 6 months; 2 - 3x/week)  N = 60  - I n = 30  50% male  42.7 (±13.3)  - C n = 30  56.7% male  46.8 (±13.4) | I: 12 weeks, after dialysis, home-based PMR involving three training sessions, provided by a researcher, followed by home exercises (20 min, 2x/day) C: Usual care  Assessments: - Pre-intervention - Post-intervention | FSS | ↓ within I d = -1.18, 95% CI [-1.73, -0.633] ≈ within C d = 0.05, 95% CI [-0.459, 0.553] I ↓ C d = -1.14, 95% CI [-1.684, -0.593] | Unclear, sample size achieved n = 12 per group | Moderate |
| Güler, 2023, Turkey [7] | RCT  HD patients (dialysis ≥ 6 months; 3x/week)  N = 58  - I n = 31  45.4% male  Age NR  - C n = 27  44.4% male  Age NR | I: 4 weeks, during dialysis, footbath with hot water (40-42 °C) (30 min, 3x/week, 12 sessions total) C: Usual care  Assessments: - Pre-intervention - Post-intervention | Fatigue VAS | ↓ within I d = -2.72, 95% CI [-3.416, -2.033] ≈ within C d = -0.28, 95% CI [-0.819, 0.253] I ↓ C d = -1.50, 95% CI [-2.081, -0.914] | A priori 0.99, sample size n = 58 in total | Weak |
| Habibzadeh, 2020, Iran [8] | RCT  HD patients (dialysis ≥ 6 months; 3x/week)  N = 120  - I1 n = 30  100% male  Age NR  - I2 n = 30  100% male  Age NR  - I3 n = 30  100% male  Age NR  - C n = 30  100% male  Age NR | I1: 8 weeks, during dialysis, foot massage with 3 cc chamomile oil, provided by a trained researcher (20 min (10 min per foot), 3x/week)  I2: 8 weeks, during dialysis, foot massage with 3 cc almond oil, provided by a trained researcher (20 min (10 min per foot), 3x/week)  I3: 8 weeks, during dialysis, foot massage without oil, provided by a trained researcher (20 min (10 min per foot), 3x/week)  C: Usual care  Assessments: - Pre-intervention - 2 months post-intervention | FSS | ↓ within I1 d = -0.54, 95% CI [-1.058, -0.027] ↓ within I2 d = -1.44, 95% CI [-2.008, -0.872] ↓ within I3 d = -0.58, 95% CI [-1.094, -0.061] ≈ within C d = -0.02, 95% CI [-0.523, 0.489] | A priori 0.8, n = 25 per group | Strong |
| Hadadian, 2016, Iran [9] | CCT  HD patients (dialysis ≥3 months)  N = 56  - I n = 28  71.4 % male  48.2 (±15.5)  - C n = 28  64.3% male  56.0 (±14.6) | I: 5 weeks, during dialysis, acupressure, three acupoints (ST36, SP6, LI4) using electrical impulses (Frequency 2.5 Hz, intensity 500 micro amps into 500 Ohms) for 50s per acupoint (5 min, 2- 3x/week, 10 sessions in total) C: 5 weeks, during dialysis, sham acupressure (avoiding placing the pseudo electrodes on true meridians or acupoints of ST36, SP6, LI4 using electrical impulses (Frequency 2.5 Hz, intensity 500 micro amps into 500 Ohms) for 50s per acupoint (5 min, 2 - 3x/week, 10 sessions in total)   Assessments: - Pre-intervention - Post-intervention | BFI | ↓ within I p < 0.001 ≈ within C p = 0.731 I ↓ C p = 0.002 | NR | Strong |
| Hadadian, 2018, Iran [10] | CCT  HD patients (dialysis ≥ 6 months)  N = 65  - I n = 27  % male NR  Age NR  - C n = 38  % male NR  Age NR | I: 4 weeks, during dialysis and home-based, PMR involving two consecutive training sessions and home-based relaxation exercises (2x/day)  C: Usual care  Assessments: - Pre-intervention - Post-intervention | BFI | ≈ within I p = 0.179 ↓ within C p = 0.027 I ↓ C p = 0.008 | A priori 0.9, sample size n = 20 per group | Weak |
| Kaplan Serin, 2020, Turkey [11] | RCT  HD patients (dialysis ≥ 6 months)  N = 96  - I n = 48  56.2% male  Age 39.1 (±15.3)  - C n = 48  75.0% male  Age 49.8 ( 14.1) | I: 6 weeks, during dialysis and home-based, PMR including an exercise handbook and CD with breathing and progressive relaxation exercises, provided by a trained researcher (30-40 min, 1x/day)  Assessments: - Pre-intervention - Post-intervention | PFS | ↓ within I d = -1.10, 95% CI [-1.525, -0.667] ↑ within C d = 0.67, 95% CI [0.255, 1.077] I ↓ C d = -2.26, 95% CI [-2.774, -1.749] | A priori 0.92,  sample size NR | Weak |
| Kesik, 2023, Turkey [12] | RCT  HD patients (dialysis ≥ 6 months; 3x/week)  N = 69  - I1 n = 23  52.2% male  Age 53.4 (±6.9)  - I2 n = 23  47.8% male  Age 53.1 (±6.9)  - C n = 23  43.5% male  Age 53.3 (±7.1) | I1: 4 weeks, during dialysis, warm thermal gel compresses (38-39 °C) on lower leg (2x 15 min, 3x/week) I2: 4 weeks, during dialysis, cold thermal gel compresses (15-16 °C) on lower leg (2x 15 min, 3x/week) C: 4 weeks, during dialysis, placebo thermal gel compresses (24-25 °C) on lower leg (2x 15 min, 3x/week)   Assessments: - Baseline - After each dialysis session (1–12) - 1 week post-intervention | PFS | After dialysis session 1–12  ↓ within I1 d = -3.06, 95% CI [-3.915, -2.211]  ↓ within I2 d = -2.15, 95% CI [-2.872, -1.421]  ↑ within C d = 1.42, 95% CI [0.775, 2.068]  1 week post-intervention  ↓ within I1 d = -1.74, 95% CI [-2.417, -1.064]  ↓ within I2 d = -1.10, 95% CI [-1.722, -0.481]  ↑ within C d = 1.55, 95% CI [0.888, 2.205] at | Post hoc 0.98,  sample size n = 69 in total | Weak |
| Lazarus, 2021, India [13] | RCT  HD patients (dialysis 3x/week)  N = 200  - I n = 100  59.0% male  Age NR  - C n = 100  68.0% male  Age NR | I: 8 weeks, during dialysis, lower back and lower leg olive oil massage (every hour/4 hours, 3x/week) C: Usual care  Assessments: - Pre-intervention - Post-intervention | FSS | ↓ within I d = -0.54, 95% CI [-0.822, -0.257] ≈ within C d = -0.22, 95% CI [-0.495, 0.061] I ↓ C d = -0.36, 95% CI [-0.635, -0.076] | A priori 0.8, sample size n = 100 per group) | Weak |
| Ozdemir, 2013, Turkey [14] | RCT  HD patients (dialysis ≥ 6 months; 3x/week)  N = 80  - I n = 40  32.5% male  Age 43.1 (±15.8)  - C n = 40  42.5% male  Age 54.0 (±12.8) | I: 1 week, during dialysis, foot reflexology, performed by a certified researcher (30 min (15 min per foot), 3x/week) C: Usual care  Assessments: - Pre-intervention - Post-intervention | PFS | ↓ within I d = -1.38, 95% CI [-1.866, -0.891] ≈ within C d = -0.00, 95% CI [-0.438, 0.438] I ≈ C d = -1.52, 95% CI [-2.014, -1.019] | NR | Weak |
| Sabouhi, 2013, Iran [15] | RCT  HD patients (dialysis ≥ 3 months)  N = 96  - I n = 32  56.3% male  Age 53.4 (±13.9)  - C1 n = 32  56.3% male  Age 55.4 (±11.5)  - C2 n = 32  56.3% male  Age 54.3 (±13.4) | I: 4 weeks, during dialysis, acupressure, six acupoints (K1, GB34, ST36, SP6, BL23, HT7), performed by a trained researcher and co-researcher (20 min, 3x/week) C1: 4 weeks, during dialysis, placebo acupressure, six acupoints (1 cm distance from K1, GB34, ST36, SP6, BL23, HT7), performed by a trained researcher and co-researcher (20 min, 3x/week) C2: Usual care  Assessments:  - Pre-intervention - Post-intervention | PFS | ↓ within I d = -1.95, 95% CI [-2.544, -1.354] ↓ within C1 d = -0.77, 95% CI [-1.276, -0.261] ≈ within C2 d = -0.09, 95% CI [-0.581, 0.399] | NR | Weak |
| Shahdadi, 2016, Iran [16] | CCT  HD patients (dialysis ≥ 6 months; 3x/week)  N = 52  - I n = 26  80.8% male  Age 47.4 (±12.5)  - C n = 26  57.7% male  Age 47.0 (±10.6) | I: 3 weeks, during dialysis, slow back massage, performed by a trained nurse (10 min, 2x/week) C: Usual care  Assessments:  - Pre-intervention - Post-intervention | FSS | ↓ within I d = -1.31, 95% CI [-1.907, -0.709] ≈ within C d = -0.11, 95% CI [-0.654, 0.434] I ↓ C d = -1.07, 95% CI [-1.648, -0.486] | NR | Moderate |
| Sharifi, 2018, Iran [17] | RCT  HD patients (dialysis ≥ 6 months, 3–4x/week)  N = 88  - I n = 45  66.7% male  Age 47.9 (±14.0)  - C n = 43  46.5% male  Age 50.1 (±16.4) | I: 30 min, during dialysis, foot reflexology, performed by a trained researcher (30 min (15 min per foot), 3 sessions/5 days) C: Usual care  Assessments:  - Baseline - After 1st session - Before and after 2nd session - Before and after 3rd session | FSS | Baseline - after 1st session  ↓ within I d = -0.58, 95% CI [-1.005, -0.161]  ≈ within C d = -0.03, 95% CI [-0.449, 0.397]  I ≈ C d = -0.31, 95% CI [-0.733, 0.108]  Before and after 3rd session ↓ within I d = -0.54, 95% CI [-0.964, -0.122]  ≈ within C d = -0.04, 95% CI [-0.467, 0.378]  I ↓ C d = -0.66, 95% CI [-1.088, -0.229] | A priori 0.9, sample size n = 43 per group | Strong |
| Suandika, 2023, Indonesia [18] | RCT  HD patients (dialysis ≥3 months)  N = 96  - I n = 49  43.4% male  Age NR  - C n = 47  52.8% male  Age NR | I: 4 weeks, during dialysis, acupressure, three acupoints (K1, ST36, SP6), performed by a trained researcher, 3 min per acupoint (18 min in total, 3x/week) C: 4 weeks, during dialysis, sham acupressure, 3.33 cm distance from three acupoints (K1, ST36, SP6), performed by a trained researcher, 3 min per acupoint (18 min in total, 3x/week)  Assessments:  - Pre-intervention - Post-intervention | BFI | I ↓ C p<0.001 | A priori 0.8, sample size n = 44 per group | Strong |
| Tsai, 2018, Taiwan [19] | RCT  HD patients (dialysis ≥ 3 months)  N = 27  - I n = 14  42.9% male  Age 62.3 (±4.8)  - C n = 13  16.7% male  Age 59.5 (±9.4) | I: 4 weeks, during dialysis, herbal acupoint therapy, patches placed on two points (K11, CV4) (4 hours, 3x/week) C: 4 weeks, during dialysis, sham herbal acupoint therapy, patches with brown clay placed on two points (K11, CV4) (4 hours, 3x/week)  Assessments:  - Pre-intervention - Post-intervention | Fatigue VAS | ↓ within I p = 0.01 ≈ within C p = 0.08 I ↓ C p = 0.001 | NR | Moderate |
| Zeb, 2023, Pakistan [20] | Cohort (one group pre post)  HD patients (dialysis ≥ 3 months; 2x/week)  N = 60  70.0% male  Age NR | I: 20 min, after dialysis, foot bath with hot water (40-43 ºC), provided by two nurses and two assistants (20 min, 1x)  Assessments: - Pre-intervention - Post-intervention | PFS | ↓ within I d = -1.53, 95% CI [-1.933, -1.120] | Unclear,  sample size n = 60 in total | Weak |
| **Exercise Interventions** | | | | | | |
| Chang, 2010, Taiwan [21] | CCT  HD patients (dialysis ≥ 3 months)  N = 71  - I n = 36  72% male  Age 50.8 (±10.7)  - C n = 35  68.6% male  Age 52.0 (±8.7) | I: 8 weeks, during dialysis, leg ergometry exercise, 12-13 RPE on Borg 6-20 scale (1st session 10 min, 2nd session 20 min, other sessions 30 min, 3x/week) C: Usual care  Assessments: - Baseline - 4 weeks after baseline - Post-intervention | HPFS | Post-intervention ↓ within I p < 0.001  ≈ within C p = 0.15 | A priori 0.7, sample size n = 30 per group | Strong |
| Cho, 2014, South Korea [22] | CCT  HD patients (dialysis 3x/week)  N = 46  - I n = 24  62.5% male  Age 60.8 (±6.9)  - C n = 24  54.2% male  Age 57.7 (±9.5) | I: 8 weeks, before dialysis (onsite gym), VR exercise program, provided by a physical therapist (40 min, 3x/week) Stretching exercises, eight positions, intensity 8-9 RPE, 5-10 sec, two repetitions (5 min). Muscle strengthening exercise games (2) and yoga exercise games (2), intensity RPE week 1&2 10-11, week 3 to 5 12-13, week 6 to 8 14, 2-3 repetitions (30 min) C: Usual care  Assessments: - Pre-intervention - Post-intervention | Fatigue VAS | ↓ within I d = -1.87, 95% CI [-2.565, -1.179] ≈ within C d = -0.09, 95% CI [-0.669, 0.488] I ↓ C d = -1.56, 95% CI [-2.21, -0.917] | A priori 0.95, sample size n = 40 in total | Weak |
| Chou, 2020, Taiwan [23] | Cohort (one group pre post)  HD patients (dialysis ≥ 3 months, 3x/week) N = 64 - I n = 32  56.3% male  Age 58.0 (±15.8) - C n = 32  56.3% male  Age 60.6 (±10.7) | I: 4 weeks, hospital based, game based (Nintendo Wii) training involving boxing (pitching, swinging, punching exercises), guided by a trained research nurse (30 min, 3x/week) C: Usual care  Assessments: - Pre-intervention - Post-intervention | NFSHD | ↓ within I d = -1.79, 95% CI [-2.371, -1.211] ↓ within C d = -1.42, 95% CI [-1.965, -0.869] I ↓ C d = -4.24, 95% CI [-5.126, -3.359] | A priori 0.8, sample size n = 32 per group | Moderate |
| Grigoriou, 2021, Greece [24] | Cohort (one group pre post)  HD patients (dialysis≥ 6 months)  N = 20  80.0% male  Age 59.0 (±13.7) | I: 36 weeks, during dialysis, aerobic and resistance exercise, provided by two trained clinical exercise scientists (60-80 min, 3x/week). Aerobic exercise: adapted bicycle ergometer, intensity 50-60% maximal exercise capacity and/or 14-16 RPE. Resistance exercises; resistance bands, ankle weights, and dumbbells for the upper and lower body, intensity 14-16 RPE  Assessments: - Pre-intervention - Post-intervention | FSS  MFI-20 BFI | FSS ≈ within I d = -0.07, 95% CI [-0.694, 0.546]  MFI ≈ within I d = -0.62, 95% CI [-1.255, 0.014]  BFI  ≈ within I d = 0.27, 95% CI [-0.351 ,0.895] | A priori 0.8, sample size n = 20 in total | Weak |
| Kumar, 2022, Pakistan [25] | RCT  HD patients (dialysis ≥ 3 months; 3x/week)  N = 26  - I n = 13  73.3% male  Age 46.1 (±10.6)  - C n = 13  73.3% male  Age 43.6 (±11.2) | I: 6 weeks, home-based, aerobic and resistance exercise (3x/week). Aerobic: walking, intensity 40-60% peak heart rate (20-30 min, 3x/week). Resistance exercise: upper and lower body muscle groups using Thera-band, intensity 50% of one repetition maximum, repetitions 1x10 (3x/week) C: Usual care  Assessments: - Pre-intervention - Post-intervention | FAS | ≈ within I d = -0.43, 95% CI [-1.152, 0.298] ≈ within C d = -0.02, 95% CI [-0.739, 0.692] I ↓ C d = -2.15, 95% CI [-3.053, -1.254] | NR | Weak |
| Malagoni, 2008, Italy [26] | CCT  HD patients (dialysis ≥ 1 year, 3x/week)  N = 20  - I n = 13  77.0% male  Age 62.0 (±10.0)  - C n = 7  57.0% male  Age 66.0 (±14.0) | I: 24 weeks, dialysis unit and home-based. Aerobic exercise: personalized walking program, intensity 50% below max treadmill speed, speed 63-80 steps/min (10min, 2x/day) C: Usual care  Assessments: - Baseline - 4 weeks after baseline - 8 weeks after baseline  - 12 weeks after baseline - 16 weeks after baseline - Post-intervention | 5 point Likert scale on self-reported Post dialysis fatigue | ≈ within I d = -0.33, 95% CI [-1.107, 0.441] ≈ within C d = -0.00, 95% CI [-1.048, 1.048] I ≈ C d = -0.21, 95% CI [-1.128, 0.715] | NR | Weak |
| Maniam, 2014, Malaysia [27] | CCT  HD patients (dialysis ≥ 6 months)  N = 47  - I n = 20  60.0% male  Age 56.3 (±8.5)  - C n = 27  51.9% male  Age 57.9 (±8.9) | I: 12 weeks, before dialysis, flexibility (stretching muscle groups; 10 sec, 5 repetitions) and resistance exercises (intensity 12-13 RPE; 30 min), provided by a qualified physical trainer in the first sessions (30-40 min, 3x/week).  C: Usual care  Assessments: - Pre-intervention - Post-intervention | FACIT-F | ↓ within I d = -1.10, 95% CI [-1.768, -0.438] ≈ within C d = 0.25, 95% CI [-0.787, 0.284] I ↓ C d = -0.99, 95% CI [-1.603, -0.379] | NR | Moderate |
| Motedayen, 2014, Iran [28] | CCT  HD patients (dialysis ≥ 3 months, 3x/week)  N = 66  - I n = 33  66.7% male  Age NR  - C n = 33  48.5% male  Age NR | I: 8 weeks, during dialysis, flexibility and resistance exercises, involving deep breathing between exercise intervals, provided by a senior expert in physical education (20 min, 2x/week) Strength and repetition extended to subjects capabilities C: Usual care  Assessments: - Baseline - 4 weeks after baseline  - Post-intervention | FSS | ↓ within I p < 0.001 ≈ within C p = 0.08 | NR | Moderate |
| Neethu, 2018, India [29] | Cohort (one group pre post)  HD patients (not specified)  N = 30  % male NR  Age NR | I: 2 weeks, during dialysis, resistance exercises involving quadriceps knee and gluteal strengthening (30 min, 2x/week)  Assessments: - Pre-intervention - Post-intervention | MAF | ↓ within I d = -3.86, 95% CI [-4.718, -3.005] | NR | Weak |
| Palar, 2022, India [30] | RCT  HD patients (dialysis ≥ 3 months)  N = 35  - I n = 19  89.5% male  Age NR  - C n = 16  50.0% male  Age NR | I: 12 weeks, during dialysis (unit), resistance exercises involving range of motion and exercises for upper and lower extremities (25 min, 2x/week) C: Usual care  Assessments: - Baseline - 7 days after baseline - 15 days after baseline - 4 weeks after baseline - Post-intervention | MFI-20 | General fatigue  ↓ within I d = -1.70, 95% CI [-2.444, -0.959]  ≈ within C d = 0.29, 95% CI [-0.411, 0.982]  I ↓ C d = -1.39, 95% CI [-2.125, -0.645]  Physical fatigue  I ↓ C d = -1.39, 95% CI [-2.125, -0.645] ↓ within I d = -1.67, 95% CI [-2.404, -0.928]  ≈ within C d = 0.35, 95% CI [-0.344, 1.053]  I ↓ C d = -0.94, 95% CI [-1.635, -0.235]  Mental fatigue ↓ within I d = -1.31, 95% CI [-2.005, -0.605]  ≈ within C d = 0.08, 95% CI [-0.614, 0.772]  I ↓ C d = -0.98, 95% CI [-1.682, -0.275] | NR | Weak |
| Paravan, 2017, Iran [31] | RCT  HD patients (dialysis ≥ 6 months)  N = 46  - I n = 23  21.7% male  Age 89.0 (±9.5)  - C n = 23  21.7% male  Age 60.2 (±10.5) | I: 8 weeks, during dialysis, aerobic exercise: pedal bicycle, intensity 30-60 RPM, provided by medical doctors specialized in exercise physiology (30-60 min, 3x/week) C: Usual care  Assessments: - Pre-intervention - Post-intervention | FSS | ↓ within I d = -0.68, 95% CI [-1.278, -0.089] ≈ within C d = -0.04, 95% CI [-0.618, 0.538] I ↓ C d = -0.61, 95% CI [-1.201, -0.018] | A priori 0.9, sample size n = 17 per group | Moderate |
| Rakocevic-Hrnjak, 2018, Serbia [32] | CCT  HD patients (dialysis ≥ 1 year)  N = 124  - I n = 54  46.3% male  Age NR  - C n = 70  63.0% male  Age NR | I: 3 years, after dialysis, kinesitherapy with ELF-PEMF (18 Hz, 2 mT); active and passive-assisted exercises per segments, 2 series, 10 repetitions, provided by a trained physiotherapist, after 10 consecutive dialysis procedures (40 min) C: Usual care  Assessments: - Baseline - 1 year after baseline - 2 years after baseline - Post-intervention | FACIT-F | ↓ within I d = -1.90, 95% CI [-2.358, -1.448] ≈ within C d = -0.12, 95% CI [-0.451, 0.212] I ↓ C d = -1.49, 95% CI [-1.895, -1.093] | NR | Strong |
| Salehi, 2020, Iran [33] | RCT  HD patients  ≥ 3 months dialysis; HD 2x/week for 4 hours  N = 37  - I n = 20  65.0% male  Age 57.8 (±9.2)  - C n = 17  76.5% male  Age 54.7 (±10.0) | I: 12 weeks, during dialysis, aerobic exercise: electric exercise bike, intensity 30 RPM (20 min, 2x/week) C: Usual care  Assessments: - Baseline - 4 week after baseline - 8 week after baseline - Post-intervention - 4 weeks post-intervention | MFI-20 | Post-intervention  ≈ within I d = -0.30, 95% CI [-0.925, 0.321]  ≈ within C d = 0.53, 95% CI [-0.156, 1.212]  I ↓ C d = -1.20, 95% CI [-1.913, -0.497]  4 weeks post-intervention  ≈ within I d = -0.32, 95% CI [-0.939, 0.308]  ≈ within C d = 0.61, 95% CI [-0.076, 1.311]  I ↓ C d = -1.43, 95% CI [-2.151, -0.703] | A priori 0.8, sample size n = 16 per group | Moderate |
| Samuel Raj, 2023, India [34] | RCT  HD patients (not specified)  N = 30  - I n = 15  % male NR  Age NR  - C n = 15  % male NR  Age NR | I: 36 weeks, during dialysis and home-based, aerobic (cycle ergometer, 5-20 min, 2x/week), resistance (resistance bands, intensity 50-90 MPR, 1 set, 2x/week), and flexibility exercises (self-stretching upper and lower limb and AROM, 5 repetition 1 set, 2x/week, guided by qualified physiotherapists (7x/week). Home exercises on non-dialysis days (5x/week) C: 36 weeks, during dialysis, usual care and hand grip exercises (2x/week)  Assessments: - Baseline - 12 weeks after baseline - 24 weeks after baseline - Post-intervention | FAS | I ↓ C p = 0.001 at 24 weeks after baseline and post-intervention | NR | Moderate |
| Wilkinson, 2019, UK [35] | RCT  Non-dialysis patients (CKD stages 3b–5)  N = 36  - I1 n = 18  39.0% male  Age 63.7 (±8.5)  - I2 n = 18  39.0% male  Age 59.6 (±14.4) | I1: 12 weeks, hospital gym, aerobic exercises (treadmill, intensity 70 - 80% MHR (30 min, 3x/week)  I2: 12 week, hospital gym, aerobic (treadmill, intensity 70-80% MHR, 20 min, 2x/week) and resistance exercises (leg extension and leg press machine, intensity 70% MHR 1x repetition, 3x 8 - 12 repetitions (10 min, 2x/week)   Assessments: - Pre-intervention - Post-intervention | FACIT-F | ↓ within I1 d = -0.69, 95% CI [-1.361, -0.016] ≈ within I2 d = -0.67, 95% CI [-1.339, 0.004] I1 ↓ I2 d = -0.93, 95% CI [-1.617, -0.242] | A priori 0.8, sample size n = 21 per group | Moderate |
| Zhou, 2023, China [36] | Interrupted time series  HD patients (dialysis ≥ 3 months, 3x/week)  N = 75  46.7% male  Age 55.0 (±10.7) | I: 12 weeks, during dialysis, aerobic (upper limb and lower limbs, 4 sets, 8 repetitions. 8-10 min, 3x/week) and resistance exercises (upper limb and lower limbs with loop bands, 5-50 lbs, 2.27 - 22.68 kg, 5-10 sets, 8-10 repetitions, 10 - 15 min, 3x/week)  Assessments: - Pre-intervention - Post-intervention - 12 weeks post-intervention | MFI-20 | Post-intervention  ≈ within I d = -0.19, 95% CI [-0.506, 0.136]  ≈ within I d = -0.32, 95% CI [-0.640, 0.004] | NR | Weak |
| **Mind-body Interventions** | | | | | | |
| Bhuvaneswari, 2020, India [37] | Cohort (one group pre post)  HD patients (not specified)  N = 60  66.7% male  Age NR | I: 15 days, after dialysis, Pranayama (30 min, 1x/day)  Assessments: - Pre-intervention - Post-intervention | FSS | ↓ within I d = -4.85, 95% CI [-5.852, -3.844] | NR | Weak |
| Bro, 2022, Denmark [38] | RCT  HD patients (not specified)  N = 19  - I n = 9  66.7% male  Age 65.0 (±20.0)  - C n = 10  90% male  Age 61.0 (±14.0) | I: 6 weeks, during dialysis, music therapy (instrumental music performed by professional acoustic guitarists, 30 min, 3x/week) C: Usual care  Assessments VAS: - Pre-intervention HD session - Post intervention HD session  Assessments MFI: - Pre-intervention - 1 week post-intervention | Fatigue VAS  MFI-20 | Fatigue VAS ≈ within I d = -0.46, 95% CI [-1.393, 0.478] ≈ within C d = 0.17, 95% CI [-0.184, 0.489] I ↓ C d = -1.02, 95% CI [-1.973, -0.059]  MFI-20 I ≈ C p > 0.05 | NR | Moderate |
| Damayanti, 2023, Indonesia [39] | CCT  HD patients (dialysis ≥3 months)  N = 42  - I n = 21  57.1% male  Age NR  - C n = 21  52.4% male  Age NR | I: 30 min, during dialysis, music therapy (natural sound by mp3 player and headset in a prone sleeping position, provided by the researcher, 1x) C: Usual care  Assessments: - Pre-intervention - Post-intervention | FACIT-F | ↓ within I d = -3.56, 95% CI [-4.536, -2.591] ↑ within C d = 2.02, 95% CI [1.28, 2.767] I ↓ C d = -24.75, 95% CI [-30.082, -19.426] | NR | Weak |
| Elahi, 2022, Iran [40] | RCT  HD patients (not specified)  N = 68  - I n = 31  67.7% male  Age 49.1 (±14.7)  - C n = 37  67.6% male  Age 50.5 (±11.4) | I: 4 weeks, home-based, aromatherapy (attaching three drops of R. damascena oil on a cotton piece attached to the collar before sleep, removed upon waking, daily reminder call, every night, 7x/week) C: Usual care  Assessments: - Pre-intervention - Post-intervention | FSS | ↓ within I d = -1.92, 95% CI [-2.519, -1.316] ≈ within C d = 0.20, 95% CI [-0.253, 0.661] I ↓ C d = -1.49, 95% CI [-2.028, -0.952] | A priori 0.9, sample size n = 37 per group | Moderate |
| Gross, 2017, USA [41] | RCT  HD patients (eligible for kidney or kidney-pancreas transplant)  N = 55  - I n = 27  29.6% male  Age NR  - C n = 28  57.1% male  Age NR | I: 8 weeks, dialysis unit and home-based, telephone-adapted mindfulness based stress reduction (meditation and yoga exercises), involving in-person, 3 hours training workshops in week 1 & 8, and 1.5 hours group teleconferences (including teacher led meditations and discussions) in week 2-7. Final workshop included an in-person retreat, provided by a certified MBSR teacher (2.5 hours, 8 sessions) C: 8 weeks, telephone-based support group, involving a bookend format with 2x 1.5 hours workshops and 6x 1 hours weekly teleconferences (building interpersonal communications skills and accessing reliable information from the internet) provided by a trained group facilitator  Assessments: - Pre-intervention - Post-intervention - 6 months post-intervention | PROMIS – F | ≈ within I d = 0.10, 95% CI [-0.456, 0.656] ≈ within C d = -0.12, 95% CI [-0.655, 0.414] I ≈ C d = -0.15, 95% CI [-0.714 - 0.409] | A priori 0.8, sample size n = 51 in total | Weak |
| Haghi, 2019, Iran [42] | Cohort (one group pre post)  HD patients (not specified)  N = 25  48.0% male  Age NR | I: 4 weeks, during dialysis, music therapy (listening to classic and modern music and two motivating compositions), headphones provided (30 min, 3x/week)  Assessments: - Pre-intervention - Post-intervention | FSS | ≈ within I d = -0.36, 95% CI [-0.919, 0.199] | A priori 0.8, sample size n = 25 in total | Weak |
| İlter, 2023, Turkey [43] | RCT  HD patients (not specified)  N = 91  - I1 n = 30  40.0% male  Age NR  - I2 n = 30  30.0% male  Age NR  - C n = 31  41.9% male  Age NR | I1: 12 weeks, during dialysis and home based, Nadi Shoshana Pranayama (video with procedure steps provided, 15-20 min, 1x/day) I2: 12 weeks, during dialysis and home-based, pursed-lip breathing (holding breath for 1-2 sec longer than breathing in, video with procedure steps provided, 15-20 min, 1x/day) C: Usual care  Assessments VAS: - Before each session - 3 hours after each session  Assessments PFS: - Pre-intervention - Post-intervention | PFS Fatigue VAS | PFS ↓ within I1d = -0.70, 95% CI [-1.225, -0.182] ≈ within I2 d = -0.14, 95% CI [-0.65, 0.363] ≈ within C d = -0.16, 95% CI [-0.658, 0.339]  Fatigue VAS ↓ within I1 d = -0.56, 95% CI [-1.077, -0.046] ≈ within I2 d = -0.40, 95% CI [-0.911, 0.111] ≈ within C d = 0.10, 95% CI [-0.401, 0.595] | A priori 0.8, sample size n = 28 per group | Weak |
| Kang, 2021, South Korea [44] | CCT  HD patients (not specified)  N = 54  - I n = 26  69.2% male  Age 60.4 (±2.4)  - C n = 28  78.6% male  Age 56.9 (±2.7) | I: 4 weeks, during dialysis, forest therapy stimuli including visual and auditory (nature video's and sounds), olfactory (cotton pad with drops of cypress oil placed on shoulder), tactile (paulownia scaffold applied) and motor (electric leg exercise machine, speed 15 steps), provided by a researcher (15 min, 3x/week) C: Usual care  Assessments: - Pre-intervention - Post-intervention | VAS-F (adjusted) | ≈ within I d = -0.24, 95% CI [-0,786, 0,305] ≈ within C d = 0.37, 95% CI [-0,163, 0,893] I ↓ C d = -0.56, 95% CI [-1.103, -0.015] | A priori 0.8, sample size n = 26 per group | Moderate |
| Karadag, 2019, Turkey [45] | CCT  HD patients ≥ 6 months dialysis  N = 60  - I n = 30  54.2% male  Age 55.8 (±13.2)  - C n = 30  45.8% male  Age 46.4 (±14.2) | I: 4 weeks, before dialysis, aromatherapy (inhaling 2% lavender oil on a gauze dressing, placed 10 cm from the nose with normal breathing, 20 min, 2-3x/ week) C: Usual care  Assessments: - Pre-intervention - Post-intervention | FSS | ↓ within I d = -0.85, 95% CI [-1.379, -0.322] ≈ within C d = 0.09, 95% CI [-0.417, 0.596] I ≈ C d = -0.44, 95% CI [-0.947, 0.077] | A priori 0.8, sample size n = 30 per group | Moderate |
| Muz, 2017, Turkey [46] | RCT  HD patients (dialysis ≥ 3 months, 3x/week) N = 62 - I n = 27  66.7% male  Age 52.3 (±14.5) - C n = 35  45.7% male  Age 59.3 (±12.3) | I: 4 weeks, home-based, aromatherapy involving lavender and orange oils (1:1 ratio) on a gauze bandage, placed 5 cm below the nose (2 min, 1x/day, before sleeping) C: Usual care  Assessments: - Baseline - 1 week after baseline  - 2 weeks after baseline - 3 weeks after baseline - Post-intervention | Fatigue VAS  PFS | Fatigue VAS ↓ within I p < 0.001 ↑ within C p = 0.002 I ↓ C p < 0.001  PFS  ↓ within I d = -3.05, 95% CI [-3.836, -2.267]  ≈ within C d = 0.10, 95% CI [-0.365, 0.573]  I ↓ C d = -2.59, 95% CI [-3.263, -1.908] | Post hoc 0.99,  sample size n = 62 in total | Moderate |
| Picariello, 2021, UK [47] | RCT  HD patients (dialysis ≥ 3 months)  N = 46  - I n = 23  21.7% male  Age 89.0 (±9.5)  - C n = 23  21.7% male  Age 60.2 (±10.5) | I: 4-6 weeks, during dialysis, CBT (self-management aimed at fatigue), provided face-to-face and by phone by a trained researcher or a certified psychologist (30-60 min, 1x/week, total 3- 5 sessions) C: Usual care  Assessments: - Baseline - 3 months after baseline | CFQ | ↓ within I d = -1.62, 95% CI [-2.559, -0.675] ↓ within C d = -1.15, 95% CI [-2.146, -0.145] I ≈ C d = -0.59, 95% CI [-1.179, 0.002] | NR | Weak |
| Rahimi, 2022, Iran [48] | RCT  HD patients (dialysis ≥ 3 months, 3x/week)  N = 62  - I n = 31  53.1% male  Age 62.3 (±11.0)  - C n = 31  46.9% male  Age 55.3 (±13.7) | I: 4 weeks, during dialysis, aromatherapy (two drops lavender oil (7% diluted in sweet almond oil) applied to a gauze and placed 20 cm from the nose, provided by a researcher, 2 min, 3x/week)  C: 4 weeks, during dialysis, placebo aromatherapy (two drops of almond oil (odorless) applied to a gauze and placed 20 cm from the nose, provided by a researcher, 2 min, 3x/week)  Assessments: - Baseline - 1 week after baseline - 2 weeks after baseline - 3 weeks after baseline - Post-intervention | PFS | ↓ within I d = -1.48, 95% CI [-2.038, -0.915] ≈ within C d = -0.10, 95% CI [-0.602, 0.396] I ↓ C d = -1.52, 95% CI [-2.082, -0.952] | A priori 0.9, sample size n = 63 in total | Strong |
| Wu, 2014, Taiwan [49] | CCT  HD patients (dialysis  ≥ 6 months, 3x/week)  N = 172  - I n = 71  31.0% male  Age 57.1 (±10.9)  - C n = 101  55.4% male  Age 58.3 (±11.5) | I: 24 weeks, during dialysis and home-based, qigong (mindfulness techniques and relaxing postures focused on rhythmic breathing, provided by a certified qigong master on dialysis days, 10 min 3x/week, and self-practice on non-dialysis days, 10 min, 2x/day, 4 x/week) C: Usual care  Measurements: - Baseline - 8 weeks after baseline - 12 weeks after baseline - 16 weeks after baseline - 20 weeks after baseline - Post-intervention | HPFS | ↓ within I d = -0.91, 95% CI [-1.255, -0.564] ↓ within C d = -0.36, 95% CI [-0.640, -0.084] I ↓ C d = -0.50, 95% CI [-0.804, -0.188] | Post hoc 0.89,  sample size n = 172 in total | Weak |
| **Energy Healing Interventions** | | | | | | |
| Buyukbayram, 2023, Turkey [50] | RCT  HD patients (not specified)  N = 60  - I n = 30  40% male  Age 46.2 (±13.9)  - C n = 30  53.3% male  Age 56.0 (±13.3) | I: 4 weeks, during dialysis, Reiki, performed by trained researcher (30-35 min, 1x/day) C: Usual care   Assessments: - Pre-intervention - Post-intervention | PFS | ↓ within I d = -0.95, 95% CI [-1.481, -0.414] ≈ within C d = 0.04, 95% CI [-0.47, 0.542] I ≈ C d = -0.14, 95% CI [-0.651, 0.363] | NR | Weak |
| Ebrahimi, 2023, Iran [51] | RCT  HD patients (dialysis ≥ 6 months)  N = 80  - I n = 40  47.5% male  37.1 (±13.4)  - C n = 40  57.5% male  Age 42.6 (±12.4) | I: 3 hours, during dialysis, sleep hygiene education program (introduction of sleep physiology, overview of sleep disorders, training on behavioral interventions (relaxation, visualization) and strategies to improve sleep hygiene, provided face-to-face and by educational pamphlets, 40-60 min per session, 3 sessions total) C: Usual care  Assessments: - Pre-intervention - 8 weeks post-intervention | MFI-20 | ↓ within I d = -0.43, 95% CI [-0.781 , -0.005] ≈ within C d = 0.37, 95% CI [-0.068, 0.816] I ≈ C d = 0.00, 95% CI [-0.434, 0.443] | A priori 0.95, sample size n = 40 per group | Weak |
| Farragher, 2021, Canada [52] | RCT  HD patients (dialysis ≥ 3 months)  N = 22  - I n = 8  53.3% male  Age 60.0 (±15.1)  - C n = 14  66.7% male  Age 64.8 (±14.4) | I: 7-9 weeks, during dialysis, energy management (three web modules, combined with applying principles and strategies to solve 3 life participation problems, provided by a trained study coordinator, 4-6 sessions) C: 7-9 weeks, during dialysis, kidney disease management, provided by a trained study coordinator, 6-8 sessions)  Assessments: - Pre-intervention - 1 week post -intervention - 12 weeks post-intervention | FSS  MFIS SONG-HD F | FSS  Hedge's G = 0.36 at 1 week post-intervention Hedge's G = favors control at 12 week post-intervention  MFIS  Hedge's G = favors control at 1 week post-intervention Hedge's G = favors control at 12 week post-intervention  SONG-HD F  Hedge's G = 0.15 at 1 week post-intervention Hedge's G = favors control at 12 week post-intervention | A priori 0.8, sample size n = 36 in total | Weak |
| Yeşil Bayülgen, 2023, Turkey [53] | RCT  HD patients (dialysis ≥ 6 months, 3x/week)  N = 61  - I n = 30  50.0% male  Age 54.6 (±15.3)  - C n = 31  45.2% male  Age 52.5 (±15.0) | I: 4 weeks, home-based, distant Reiki, performed by a trained researcher (36-40 min, 3x/week) C: Usual care  Assessments: - Baseline - Post-intervention - 4 weeks post-intervention | FSS | Post-intervention  ↓ within I d = -1.79, 95% CI [-2.392, -1.193]  ≈ within C d = 0.16, 95% CI [-0.339, 0.658]  I ↓ C d = -1.13, 95% CI [-1.668, -0.587]  4 weeks post-intervention  ↓ within I d = -2.12, 95% CI [-2.749, -1.485]  ≈ within C d = 0.13, 95% CI [-0.369, 0.627]  I ↓ C d = -1.52, 95% CI [-2.094, -0.954] | A priori 0.8, sample size n = 23 per group | Weak |
| **Combination of Interventions** | | | | | | |
| Amini, 2016, Iran [54] | RCT  HD patients (dialysis ≥ 12 months)  N = 100  - I1 n = 33  66.7% male  Age 56.1  - I2 n = 32  65.6% male  Age 54.3  - C n = 35  60.0% male  Age 55.2 | I1: 8 weeks, home-based, aerobic exercise  I2: 8 weeks, home-based, Progressive Muscle Relaxation (daily)  C: Usual care  Assessments: - Pre-intervention - Post-intervention | PFS  RFS | PFS  ≈ within I1 d = 0.07, 95% CI [-0.617, 0.748] ↓ within I2 d = -0,56, 95% CI [-1.05, -0.066]   ≈ within C d = -0.08, 95% CI [-0.741, 0.585]  RFS ≈ within I1 d = -0.47, 95% CI [-1.168, 0.237] ↓ within I2 d = -0.94, 95% CI [-1.662, -0.224] ≈ within C d = -0.19, 95% CI [-0.85, 0.478] | NR | Weak |
| Chen, 2008, Taiwan [55] | RCT  PD patients (dialysis ≥ 3 months)  N = 26 - I n = 13  61.5% male  Age: 51.9 (±8.6) - C n = 13  53.8% male  Age 48.7 (±14.6) | I: 4 weeks, CBT (cognitive and behavioral components (sleep restriction, stimulus control, relaxation), delivered by a psychiatrist, 1x/week) and sleep hygiene education (impact lifestyle habits and environmental factors on sleep quality, 1x) C: 1 hour, sleep hygiene education (impact lifestyle habits and environ-mental factors on sleep quality, 1x)  Assessments: - Pre-intervention - Post-intervention | FSS | ↓ within I p = 0.006 ≈ within C p = 0.8 | A priori 0.8, sample size n = 20-30 in total | Moderate |
| Chen, 2011, Taiwan [56] | RCT  HD patients (dialysis  ≥ 6 months, 3x/ week)  N = 72  - I n = 37  45.9% male  Age 57.0 (±9.0)  - C n = 35  37.1% male  Age 59.0 (±11.0) | I: 6 weeks, dialysis unit, CBT (video-assisted cognitive and behavioral components with group discussion and education, provided by two psychiatrists and an assistant psychologist, 30 min, 3x/week) and sleep hygiene education (impact lifestyle habits and environmental factors on sleep quality, 1x, first week) C: 6 weeks, dialysis unit, sleep problem consultations (provided by psychiatrist or assistant psychologist, 1x/week or more depending on participant’s needs) and sleep hygiene education (impact lifestyle habits and environmental factors on sleep quality, 1x, first week)  Assessments: - Pre-intervention - Post-intervention | FSS | ↓ within I d = -0.59, 95% CI [-1.054, -0.124] ≈ within C d = 0.07, 95% CI [-0.4, 0.537] I ≈ C d = -0.18, 95% CI [-0.645, 0.282] | A priori 0.8, sample size n = 38-104 in total | Moderate |
| Eroglu, 2022, Turkey [57] | RCT  HD patient (dialysis ≥ 3 months, 2-3x/week  N = 61  - I n = 30  50% male  Age 52.0 (±15.2)  - C n = 31  64.5% male  Age 58.7 (±14.6) | I: 8 weeks, before dialysis, Benson relaxation and music therapy (deep breathing techniques while listening to nonverbal classical song, provided by the trained PI, 20 min, 2x/week) C: Usual care  Assessments: - Baseline - 4 weeks after baseline - 8 weeks after baseline - 10 week (2 weeks post-intervention) | PFS | I ↓ C p < 0.01 at week 4, 8 and 10 | A priori 0.8, sample size n = 30 per group | Moderate |
| Hassanzadeh, 2018, Iran [58] | RCT  HD patients (dialysis ≥ 3 months)  N = 105  - I1 n = 35  54.3% male  Age 42.7 (±12.4)  - I2 n = 35  45.7% male  Age 41.3 (±12.6)  - C n = 35  68.6% male  Age 44.4 (±11.5) | I1: 4 weeks, dialysis unit and home-based, Benson muscle relaxation (group training, 3x, 20 min sessions before, during and after dialysis, and audio file and training pamphlet 15-20 min, 2x/day) I2: 4 weeks, during dialysis and home-based, aromatherapy (two drops of 5% lavender essential oil inoculated in sweet almond oil on a cotton ball and attached to the collar with normal breathing, 15-20 min, 2x/day (morning and evening/or during dialysis). Subjects received training how to perform the intervention in groups, 3x, 20 min sessions before, during and after dialysis) C: Usual care  Assessments: - Pre-intervention - Post-intervention | BFI | ↓ within I1 d = -1.33, 95% CI [-1.845, -0.814] ↓ within I2 d = -2.96, 95% CI [-3.636, -2.282] ≈ within C d = -0.18, 95% CI [-0.649, 0.297] | A priori 0.8, sample size n = 26 per group | Weak |
| Huang, 2021, Taiwan [59] | RCT  HD patients (not specified)  N = 83  - I n = 40  72.5% male  Age 53.7 (±10.0)  - C n = 43  65.1% male  Age 61.2 (±10.2) | I: 12 weeks, during dialysis, resistance exercise (low-intensity leg exercises) and breathing technique (5x abdominal breathing), provided by a researcher, 15 min, 3x/week). Subjects were guided by a prepared video to practice 2x/day until exercise was performed correctly C: Usual care  Assessments: - Baseline - 4 weeks after baseline - 8 weeks after baseline - Post-intervention | NFSHD | ≈ within I d = -0.09, 95% CI [-0.525, 0.352] ≈ within C d = -0.12, 95% CI [-0.302, 0.544] I ≈ C d = -0.24, 95% CI [-0.667, 0.197] | A priori 0.8, sample size n = 76 in total | Moderate |
| Jhamb, 2023a, USA [60] | Cohort (one group pre post)  HD patients (dialysis ≥ 3 months, 3x/week)  N = 13  46% male  Age 63.6 (±15.1) | 12 weeks, during dialysis, resistance and aerobic exercise (video-based dialysis chair exercises with dumbbells, ankle weights and towel roll, intensity 12-14 RPE, assistance from the dialysis team, 45 min) and exercise education (educational video with patient testimonials and information from a physical therapist and nephrologist, educational booklet. motivational interviewing session with patient education expert (1x), and motivational video clips (5-10 min each), 45-60 min, 1x/week)  Assessments: - Pre-intervention - Post-intervention | FACIT-F | Feasibility study | NR | Weak |
| Jhamb, 2023b, USA [61] | RCT  HD patients (dialysis 3x/week)  N = 160  - I n = 83  59.0% male  Age 57.9 (±14.0)  - C n = 77  50.7% male  Age 57.8 (±13.7) | I: 12 weeks, during dialysis, digital CBT (pharmacotherapy and personalized treatment, provided by certified psychologists, 45-60 min, 1x/week) C: 12 weeks, during dialysis or home-based, telemedicine health education (educational materials, provided by trained research coordinators, 20-30 min, 1x/2weeks)  Assessments: - Pre-intervention - Post-intervention | FACIT-F | I ↓ C p = 0.01 | A priori 0.88,  sample size n = 150 in total | Moderate |
| Kao, 2012, Taiwan [62] | CCT  CKD patients (GFR ≥15 ml/min/1.73m2, in stable conditions and not on dialysis)  N = 94 - I n = 45  73.3% male  Age 71.2 (±11.0) - C n = 49  83.7% male  75.0 (±10.7) | I: 12 weeks, hospital and home-based, exercise (tailored exercise program, individual guidance by phone (1-4x month), intensity based on the "talk-test", 30 min, 3-5x/week) and health education (written materials, sharing exercise experience, and 1.5 hours general lecture about disease and exercise, exercise recommendations and exercise planning, provided by three instructors C: Usual care  Assessments: - Pre-intervention - Post-intervention | Fatigue scale | ≈ within I d = -0.27, 95% CI [-0,689, 0,141] ≈ within C d = 0.06, 95% CI [-0,342, 0,452] I ≈ C d = -0.20, 95% CI [-0.605, 0.206] | A priori 0.8, sample size n =52 in total | Moderate |
| Komariah, 2022, Indonesia [63] | CCT  HD patients (dialysis ≥ 3 months, 2x/week)  N = 40  - I n = 20  50.0% male  Age NR  - C n = 20  50.0% male  Age NR | I: 3 weeks, during dialysis, slow back massage and listening to an audio recording of the Holy Qur'an, provided by trained researchers (15 min, 2x/week)  C: Usual care  Assessments: - Pre-intervention - Post-intervention | FACIT-F | ↓ within I d = -3.01, 95% CI [-3.919, -2.108] ≈ within Cd = 0.41, 95% CI [-0.213, 1.042] I ↓ C d = -3.01, 95% CI [-3.919, -2.108] | A priori 0.8, sample size n = 20 per group | Moderate |
| Sari, 2023, Indonesia [64] | Cohort (two groups pre and post intervention)  HD patients (dialysis, ≥ 6 months)  N = 220  - I n = 110  60.0% male  Age NR  - C n = 110  72.7% male  Age NR | I: 4 weeks, during dialysis, AROM (exercises of all upper and lower joints, 3 repetitions and deep breathing exercises, provided by researchers and nursing staff, 30 min, 2x/week) C: Usual care  Assessments: - 1 day pre-intervention - 1 day post-intervention | FACIT-F | ↓ within I p < 0.001 ≈ within C p = 0.317 I ↓ C p < 0.001 | NR | Weak |
| Varaei, 2020, Iran [65] | RCT  HD patients (dialysis ≥ 1 year, 3x/week)  N = 96  - I1 n = 32  % male NR  Age NR  - I2 n = 32  % male NR  Age NR  - C n = 32  % male NR  Age NR | I1: 8 weeks, during dialysis, aroma therapy (gauze with lavender and sweet orange oils attached to the collar, 20 min, 3x/week) I2: 8 weeks, during dialysis, aroma-therapy applied by foot massage (lavender and sweet orange oil, performed by a massage therapist, 20 min, 3x/week) C: Usual care   Assessments:  - Pre-intervention - Post-intervention - 8 weeks post-intervention | RFS | 8 week after baseline  ↓ within I1 d = -0.88, 95% CI [-1.397, -0.370]  ↓ within I2 d = -1.85, 95% CI [-2.431, -1.261]  ≈ within C d = 0.34, 95% CI [-0.156, 0.831]  16 week after baseline ≈ within I1 d = -0.27, 95% CI [-0.762, 0.222]  ↓ within I2 d = -1.32, 95% CI [-1.859, -0.778]  ↑ within C d = 0.90, 95% CI [0.387, 1.416] | A priori 0.8, sample size n = 32 per group | Moderate |
| Yurtkuran, 2007, Turkey [66] | RCT  HD patients (dialysis ≥ 6 months, 3x/week)  N = 40  - I n = 20  45.0% male  Age 38.0 (±14.2)  - C n = 20  35.0% male  Age 41 (±10.0) | I: 12 weeks, dialysis and home-based, yoga (seven postures, 10 repetitions per posture, provided by a certified yoga teacher at the unit, 15 min, 2x/week first 4 weeks, 30 min, 2x/week for 6-12 weeks) and AROM exercises (at home, instructed by a physiotherapist, 10 min, 7x/week) C: 12 weeks, home-based, AROM exercises, instructed by a physiotherapist (10 min, 7x/week)  Assessments: - Pre-intervention - Post-intervention | Fatigue VAS | I ↓ C p = 0.008 | NR | Moderate |
| Zuo, 2022, China [67] | CCT  HD patients (dialysis ≥ 3 months dialysis; stable disease status)  N = 114  - I n = 60  50.0% male  Age NR  - C n = 54  59.3% male  Age NR | I: 24 weeks, dialysis and home-based, aerobic exercise (walking 6000 steps/day) and health education (behavioral self-management and motivational interviewing, 20 min, 1x/month, provided by trained nurses C: Usual care  Assessments: - Pre-intervention - Post-intervention | PFS | I ↓ C p < 0.001 | A priori 0.8, sample size n = 100 in total | Moderate |

*Note.* CCT = Controlled Clinical Trial; RCT = Randomized Controlled Trial; NR = Not reported; HD = hemodialysis; I = Intervention group; C = Control group; CBT = Cognitive behavioral therapy; PMR = Progressive muscle relaxation; RPE = Rating of perceived exertion; VR = Virtual reality; ELF-PEMF = Extremely low frequency pulsed electromagnetic field; RPM = rotations per minute; MPR = Maximum predicted resistance; MHR = Maximum heart rate; RHR = Resting heart rate; AROM = Active range of motion; MBSR = Mindfulness-based stress reduction; FSS = Fatigue Severity Scale; PFS = Piper Fatigue Scale; VAS-F = Visual Analogue Scale - Fatigue; Fatigue VAS = Fatigue Visual Analogue Scale; BFI = Brief Fatigue Inventory; HPFS = Hemodialysis Patients Fatigue Scale; NFSHD = Novel Fatigue Scale for Hemodialysis; MFI-20 = Multidimensional Fatigue Inventory; FACIT-F = Functional Assessment of Chronic Illness Therapy - Fatigue; MAF = Multidimensional Assessment of Fatigue; FAS = Fatigue Assessment Scale; PROMIS – F = Patient Reported Outcome Measurement Information System - Fatigue; CFQ = Chalder Fatigue Questionnaire; MFIS = Modified Fatigue Impact Scale; SONG-HD F = Standardized Outcomes in Nephrology - Hemodialysis Fatigue; RFS = Rhoten Fatigue Scale; d = Cohen’s d.

1. Ahmadidarrehsima S, Mohammadpourhodki R, Ebrahimi H, Keramati M, Dianatinasab M. Effect of foot reflexology and slow stroke back massage on the severity of fatigue in patients undergoing hemodialysis: a semi-experimental study. *J Complement Integr Med*. 2018;15(4)doi:10.1515/jcim-2017-0183

2. Biçer S, Taşci S. The effect of body acupressure on blood pressure and fatigue levels in individuals suffering from hypotension during hemodialysis: a randomized controlled trial. *Altern Ther Health Med*. 2022;28(2):6-16.

3. Çeçen S, Lafcı D. The effect of hand and foot massage on fatigue in hemodialysis patients: A randomized controlled trial. *Complement Ther Clin Pract*. 2021;43:101344. doi:10.1016/j.ctcp.2021.101344

4. Cho YC, Tsay SL. The effect of acupressure with massage on fatigue and depression in patients with end-stage renal disease. *J Nurs Res*. 2004;12(1):51-9. doi:10.1097/01.jnr.0000387488.63438.9a

5. Eğlence R, Karataş N, Taşci S. The effect of acupressure on the level of fatigue in hemodialysis patients. *Altern Ther Health Med*. 2013;19(6):23-31.

6. Ghozhdi M, Ghaljeh, M, Khazaei, N. The effect of progressive muscle relaxation technique on fatigue, pain and quality of life in dialysis patients: a clinical trial study. *Ev Based Care J*. 2023;12(4):7-16. doi:10.22038/EBCJ.2022.65275.2708

7. Güler S, Şahan S, Ülker T, Sipahioğlu MH. The effect of footbath applied to patients receiving hemodialysis treatment on comfort, fatigue, and dialysis symptoms: a randomized controlled study. *Ther Apher Dial*. 2024;28(1):23-33. doi:10.1111/1744-9987.14043

8. Habibzadeh H, Wosoi Dalavan O, Alilu L, Wardle J, Khalkhali H, Nozad A. Effects of foot massage on severity of fatigue and quality of life in hemodialysis patients: a randomized controlled trial. *Int J Community Based Nurs Midwifery*. 2020;8(2):92-102. doi:10.30476/ijcbnm.2020.81662.0

9. Hadadian F, Sohrabi N, Farokhpayam M, et al. The effects of transcutaneous electrical acupoint stimulation (TEAS) on fatigue in haemodialysis patients. *J Clin Diagn Res*. 2016;10(9):Yc01-yc04. doi:10.7860/jcdr/2016/19516.8532

10. Hadadian F, Jalalvandi, F., Karimi, S., Abdi, A., Salari, N., Ghobadi, A. Studying the effect of progressive muscle relaxation technique on fatigue in hemodialysis patients. *Annals of Trop Med and Public Health*. 2018;11(1)

11. Kaplan Serin E, Ovayolu N, Ovayolu Ö. The effect of progressive relaxation exercises on pain, fatigue, and quality of life in dialysis patients. *Holist Nurs Pract*. 2020;34(2):121-128. doi:10.1097/hnp.0000000000000347

12. Kesik G, Ozdemir L, Yıldırım T, Jabrayilov J, Çeliksöz G. Effects of warm or cold compresses applied to the legs during hemodialysis on cramps, fatigue, and patient comfort: a placebo-controlled randomized trial. *Hemodial Int*. 2023;27(2):117-125. doi:10.1111/hdi.13070

13. Lazarus ER, Deva Amirtharaj A, Jacob D, Chandrababu R, Isac C. The effects of an olive-oil massage on hemodialysis patients suffering from fatigue at a hemodialysis unit in southern India - a randomized controlled trial. *J Complement Integr Med*. 2020;18(2):397-403. doi:10.1515/jcim-2019-0338

14. Ozdemir G, Ovayolu N, Ovayolu O. The effect of reflexology applied on haemodialysis patients with fatigue, pain and cramps. *Int J Nurs Pract*. 2013;19(3):265-73. doi:10.1111/ijn.12066

15. Sabouhi F, Kalani L, Valiani M, Mortazavi M, Bemanian M. Effect of acupressure on fatigue in patients on hemodialysis. *Iran J Nurs Midwifery Res*. 2013;18(6):429-34.

16. Shahdadi H, Hodki, RM., Aliabadi, A., Sheikh, A., Moghadasi, A. The effect of slow stroke back massage on fatigue in patients undergoing hemodialysis: a randomized clinical trial. *Int J of Pharm & Tech*. 2016;8(3):16016-16023.

17. Sharifi S, Navidian A, Jahantigh M, Shamsoddini Lori A. Investigating the impact of foot reflexology on severity of fatigue in patients undergoing hemodialysis: a clinical trial study. *Med - Surg Nurs J*. 2018;In Press. doi:10.5812/msnj.81634

18. Suandika M, Chen SY, Fang JT, et al. Effect of acupressure on fatigue in hemodialysis patients: a single-blinded randomized controlled trial. *J Integr Complement Med*. 2023;29(2):111-118. doi:10.1089/jicm.2022.0644

19. Tsai MY, Wu CH, Huang YC, et al. Treatment of intradialytic hypotension with an herbal acupoint therapy in hemodialysis patients: a randomized pilot study. *Complement Ther Med*. 2018;38:67-73. doi:10.1016/j.ctim.2018.04.007

20. Zeb A, Iqbal, M, Aurangzeb, AN, Suliman, M, Sultan, A. Effect of warm water foot bath on post dialysis fatigue in patients on maintenance hemodialysis. *Pakistan J of Med and Health Sci*. 2023;17(4):52-54. doi:10.53350/pjmhs202317452

21. Chang Y, Cheng SY, Lin M, Gau FY, Chao YF. The effectiveness of intradialytic leg ergometry exercise for improving sedentary life style and fatigue among patients with chronic kidney disease: a randomized clinical trial. *Int J Nurs Stud*. 2010;47(11):1383-8. doi:10.1016/j.ijnurstu.2010.05.002

22. Cho H, Sohng KY. The effect of a virtual reality exercise program on physical fitness, body composition, and fatigue in hemodialysis patients. *J Phys Ther Sci*. 2014;26(10):1661-5. doi:10.1589/jpts.26.1661

23. Chou HY, Chen SC, Yen TH, Han HM. Effect of a virtual reality-based exercise program on fatigue in hospitalized Taiwanese end-stage renal disease patients undergoing hemodialysis. *Clin Nurs Res*. 2020;29(6):368-374. doi:10.1177/1054773818788511

24. Grigoriou SS, Krase AA, Karatzaferi C, et al. Long-term intradialytic hybrid exercise training on fatigue symptoms in patients receiving hemodialysis therapy. *Int Urol Nephrol*. 2021;53(4):771-784. doi:10.1007/s11255-020-02711-8

25. Kumar N, Sheraz S, Pereira FA, Razzaq A, Angela C, Saad SM. Effects of home-based exercise program on physical functioning of hemodialysis patients: A randomized controlled trial. *Healthcare in Low-resource Settings*. 2022;10(1)

26. Malagoni AM, Catizone L, Mandini S, et al. Acute and long-term effects of an exercise program for dialysis patients prescribed in hospital and performed at home. *J Nephrol*. 2008;21(6):871-8.

27. Maniam R, Subramanian P, Singh SK, Lim SK, Chinna K, Rosli R. Preliminary study of an exercise programme for reducing fatigue and improving sleep among long-term haemodialysis patients. *Singapore Med J*. 2014;55(9):476-82. doi:10.11622/smedj.2014119

28. Motedayen Z, Nehrir B, Tayebi A, Ebadi A, Einollahi B. The effect of the physical and mental exercises during hemodialysis on fatigue: a controlled clinical trial. *Nephrourol Mon*. 2014;6(4):e14686. doi:10.5812/numonthly.14686

29. Neethu M, Chitra AF. Effectiveness of intradialytic leg exercise on fatigue and activities of daily living. *Int J of Nurs Edu*. 2018;10(4):161. doi:10.5958/0974-9357.2018.00125.3

30. Palar R, Lobo D. Impact of intradialytic exercise on fatigue, biochemical and physiological parameters in patients on maintenance hemodialysis - a pilot study - part 1. *Clin Epi and Global Health*. 2022;15doi:10.1016/j.cegh.2022.101064

31. Paravan K, Jabar-Zadeh, F, Sarbakhsh, P, Akhtari-Shojai, E, Zarei, T. The effect of exercise during hemodialysis on fatigue and self-efficacy in patients: a blind randomized clinical trial. *Ann of Clin and Anal Med*. 2017;08(Suppl_5)

32. Rakocevic-Hrnjak A, Vuksanovic M, Dimkovic N, Djurovic A, Petronijevic N, Petronijevic M. Effects of extremely low frequency pulsed electromagnetic field added to kinesitherapy procedure on quality of life in patients with end stage renal disease on dialysis. *Vojnosanitetski pregled*. 2018;75(9):891-904. doi:10.2298/vsp160620007r

33. Salehi F, Dehghan M, Mangolian Shahrbabaki P, Ebadzadeh MR. Effectiveness of exercise on fatigue in hemodialysis patients: a randomized controlled trial. *BMC Sports Sci Med Rehabil*. 2020;12:19. doi:10.1186/s13102-020-00165-0

34. Samuel Raj VV, Mangalvedhe PV, Shetty MS, Balakrishnan DC. Impact of exercise on fatigue in patients undergoing dialysis in a tertiary care hospital. *Cureus*. 2023;15(2):e35004. doi:10.7759/cureus.35004

35. Wilkinson TJ, Watson EL, Gould DW, et al. Twelve weeks of supervised exercise improves self-reported symptom burden and fatigue in chronic kidney disease: a secondary analysis of the 'ExTra CKD' trial. *Clin Kidney J*. 2019;12(1):113-121. doi:10.1093/ckj/sfy071

36. Zhou L, Shi D, Zhang L, Wang Q, Chen L, Chen H. Does intradialytic group exercise programme influence patient-reported outcomes, laboratory parameters, and anthropometric parameters in maintenance hemodialysis patients? A single-group repeated-measures trial. *Patient Prefer Adherence*. 2023;17:491-501. doi:10.2147/ppa.S400005

37. Bhuvaneswari G, Deepika S. Effectiveness of Pranayama on fatigue and insomnia among patients with hemodialysis. *Int J of Research in Pharm Sci*. 2020;11(SPL4):171-175. doi:10.26452/ijrps.v11iSPL4.3764

38. Bro ML, Finderup J, Smilde R, Dreyer P, Gram B. Live music during haemodialysis: a multiple methods randomised controlled pilot study. *J Ren Care*. 2022;50:24-35. doi:10.1111/jorc.12453

39. Damayanti SF, S; Nekada, CDY; Tanda, US; Nugroho, A; Mindarsih, E. Music relaxation therapy effectively reduces fatigue in patients under hemodialysis. *Int Med J* 2023;30(1):44-47.

40. Elahi B, Parvan K, Jabbarzadeh Tabrizi F, Sarbakhsh P, Gharekhani A, Roshangar F. Effect of rosa damascena oil aromatherapy on fatigue severity in patients receiving hemodialysis: a randomized controlled trial. *Cr J of Med and Bio Sci*. 2022;9(3):147-152. doi:10.34172/cjmb.2022.25

41. Gross CR, Reilly-Spong M, Park T, Zhao R, Gurvich OV, Ibrahim HN. Telephone-adapted mindfulness-based stress reduction (tMBSR) for patients awaiting kidney transplantation. *Contemp Clin Trials*. 2017;57:37-43. doi:10.1016/j.cct.2017.03.014

42. Haghi S, Zadeh, SM., Vafayee, M. The effect of music on fatigue and anxiety of patients undergoing hemodialysis. *Int J of Cardio Prac*. 2019;28(1):20-25. doi:10.29252/anm-28014

43. İlter SM, Ovayolu Ö. The effect of pranayama applied to hemodialysis patients on fatigue: a randomized controlled trial. *Holist Nurs Pract*. 2023;37(6):318-329. doi:10.1097/hnp.0000000000000613

44. Kang H, Chae Y. Effects of Integrated Indirect Forest Experience on Emotion, Fatigue, Stress and Immune Function in Hemodialysis Patients. *Int J Environ Res Public Health*. Feb 10 2021;18(4)doi:10.3390/ijerph18041701

45. Karadag E, Samancioglu Baglama S. The effect of aromatherapy on fatigue and anxiety in patients undergoing hemodialysis treatment: a randomized controlled study. *Holist Nurs Pract*. 2019;33(4):222-229. doi:10.1097/hnp.0000000000000334

46. Muz G, Taşcı S. Effect of aromatherapy via inhalation on the sleep quality and fatigue level in people undergoing hemodialysis. *Appl Nurs Res*. 2017;37:28-35. doi:10.1016/j.apnr.2017.07.004

47. Picariello F, Moss-Morris R, Norton S, et al. Feasibility trial of cognitive behavioral therapy for fatigue in hemodialysis (BReF intervention). *J Pain Symptom Man*. 2021;61(6):1234-1246.e5. doi:10.1016/j.jpainsymman.2020.10.005

48. Rahimi E, Sedighi Chafjiri A, Hasavari F, Kazem Nezhad Leyli E, Naseri M, Khosravi M. Evaluation of the effect of lavender aroma on fatigue among hemodialysis patients. *Holist Nurs Pract*. 2022;36(2):76-84. doi:10.1097/hnp.0000000000000501

49. Wu CY, Han HM, Huang MC, Chen YM, Yu WP, Weng LC. Effect of qigong training on fatigue in haemodialysis patients: A non-randomized controlled trial. *Complement Ther Med*. 2014;22(2):244-50. doi:10.1016/j.ctim.2014.01.004

50. Buyukbayram Genc Z, Citlik Saritas S. The effect of reiki intervention on fatigue and anxiety in hemodialysis patients: a randomized controlled study. *Holist Nurs Pract*. 2024;38(1):26-31. doi:10.1097/hnp.0000000000000625

51. Ebrahimi F, Sokhtseraei S, Navidian A. The effect of sleep hygiene education on sleep quality, depression, and fatigue of hemodialysis patients. *Med -Surg Nurs J*. 2023;12(1):e138254. doi:10.5812/msnj-138254

52. Farragher JF, Ravani P, Manns B, et al. A pilot randomised controlled trial of an energy management programme for adults on maintenance haemodialysis: the fatigue-HD study. *BMJ Open*. 2022;12(2):e051475. doi:10.1136/bmjopen-2021-051475

53. Yeşil Bayülgen M, Gün M. The effect of Reiki on fatigue and comfort in hemodialysis patients. *Explore (NY)*. 2023;19(4):553-560. doi:10.1016/j.explore.2022.12.009

54. Amini E, Goudarzi, I, Masoudi, R, Ahmadi, A, Momeni, A. Effect of progressive muscle relaxation and aerobic exercise on anxiety, sleep quality, and fatigue in patients with chronic renal failure undergoing hemodialysis *Pak J Med Health Sci*. 2016;17(4)

55. Chen HY, Chiang CK, Wang HH, et al. Cognitive-behavioral therapy for sleep disturbance in patients undergoing peritoneal dialysis: a pilot randomized controlled trial. *Am J Kidney Dis*. 2008;52(2):314-23. doi:10.1053/j.ajkd.2008.03.012

56. Chen HY, Cheng IC, Pan YJ, et al. Cognitive-behavioral therapy for sleep disturbance decreases inflammatory cytokines and oxidative stress in hemodialysis patients. *Kidney Int*. 2011;80(4):415-22. doi:10.1038/ki.2011.151

57. Eroglu H, Gok Metin Z. Benson relaxation technique combined with music therapy for fatigue, anxiety, and depression in hemodialysis patients: a randomized controlled trial. *Holist Nurs Pract*. 2022;36(3):139-148. doi:10.1097/hnp.0000000000000509

58. Hassanzadeh M, Kiani F, Bouya S, Zarei M. Comparing the effects of relaxation technique and inhalation aromatherapy on fatigue in patients undergoing hemodialysis. *Complement Ther Clin Pract*. 2018;31:210-214. doi:10.1016/j.ctcp.2018.02.019

59. Huang HY, Hung KS, Yeh ML, Chou HL, Yeh AL, Liao TY. Breathing-based leg exercises during hemodialysis improve quality of life: a randomized controlled trial. *Clin Rehabil*. 2021;35(8):1175-1184. doi:10.1177/02692155211000738

60. Jhamb M, Devaraj SM, Alemairi M, et al. A comprehensive exercise (COMEX) intervention to optimize exercise participation for improving patient-centered outcomes and physical functioning in patients receiving hemodialysis: development and pilot testing. *Kidney Med*. 2023;5(11):100720. doi:10.1016/j.xkme.2023.100720

61. Jhamb M, Steel JL, Yabes JG, et al. Effects of technology assisted stepped collaborative care intervention to improve symptoms in patients undergoing hemodialysis: the taccare randomized clinical trial. *JAMA Intern Med*. 2023;183(8):795-805. doi:10.1001/jamainternmed.2023.2215

62. Kao YH, Huang YC, Chen PY, Wang KM. The effects of exercise education intervention on the exercise behaviour, depression, and fatigue status of chronic kidney disease patients. *Health Education*. 2012;112(6):472-484. doi:10.1108/09654281211275827

63. Komariah A, Rochmawati E. The effect of listening to the holy qur'an and a back massage on fatigue and quality of life for participants undergoing hemodialysis: a quasi-experimental study. *J Relig Health*. 2023;62(6):4334-4346. doi:10.1007/s10943-022-01664-9

64. Sari RY, Kartini Y, Faizah I, Rohmawati R, Hasina SN. Combination of AROM with deep breathing exercise against fatigue and quality of life of hemodialysis patients; an experimental study. *J of Nephropharma*. 2023;13(1):e10551. doi:10.34172/npj.2023.10551

65. Varaei S, Jalalian Z, Yekani Nejad MS, Shamsizadeh M. Comparison the effects of inhalation and massage aromatherapy with lavender and sweet orange on fatigue in hemodialysis patients: a randomized clinical trial. *J Complement Integr Med*. 2020;18(1):193-200. doi:10.1515/jcim-2018-0137

66. Yurtkuran M, Alp A, Yurtkuran M, Dilek K. A modified yoga-based exercise program in hemodialysis patients: a randomized controlled study. *Complement Ther Med*. 2007;15(3):164-71. doi:10.1016/j.ctim.2006.06.008

67. Zuo M, Zhu W, Lin J, et al. The impact of nurse-led nonpharmacological multidisciplinary holistic nursing care on fatigue patients receiving hemodialysis: a randomized, parallel-group, controlled trial. *BMC Nurs*. 2022;21(1):352. doi:10.1186/s12912-022-01126-3

# **Electronic Supplementary Material 3**

# *Quality Assessment*

| **First Author, Year (Country)** | **Selection Bias** | **Study Design** | **Confounders** | **Blinding** | **Data Collection Methods** | **Withdrawals and Dropouts** | **Global Rating** |
| --- | --- | --- | --- | --- | --- | --- | --- |
| Ahmadidarrehsima, 2018 (Iran) | **-/+** | **+** | **+** | **-** | **+** | **+** | **-/+** |
| Amini, 2016  (Iran) | **-/+** | **+** | **+** | **-** | **+** | **-** | **-** |
| Bhuvaneswari, 2020 (India) | **-/+** | **-/+** | **-** | **-** | **-** | **-** | **-** |
| Biçer, 2021 (Turkey) | **-/+** | **+** | **-** | **-** | **+** | **+** | **-** |
| Bro, 2022 (Denmark) | **-/+** | **+** | **+** | **-** | **+** | **-/+** | **-/+** |
| Buyukbayram, 2023 (Turkey) | **+** | **+** | **-** | **-** | **+** | **+** | **-** |
| Cecen, 2021 (Turkey) | **-/+** | **+** | **+** | **-** | **+** | **+** | **-/+** |
| Chang, 2010 (Taiwan) | **+** | **+** | **+** | **-/+** | **+** | **-/+** | **+** |
| Chen, 2008 (Taiwan) | **-/+** | **+** | **+** | **-** | **+** | **+** | **-/+** |
| Chen, 2011 (Taiwan) | **+** | **+** | **+** | **-** | **+** | **+** | **-/+** |
| Cho, 2004 (Taiwan) | **-/+** | **+** | **-** | **-** | **-** | **+** | **-** |
| Cho, 2014 (South Korea) | **-/+** | **+** | **+** | **-** | **+** | **+** | **-/+** |
| Chou, 2020 (Taiwan) | **-/+** | **+** | **+** | **-/+** | **-** | **+** | **-/+** |
| Damayanti, 2023 (Indonesia) | **-/+** | **+** | **-** | **-** | **+** | **-** | **-** |
| Ebrahimi, 2023 (Iran) | **-/+** | **+** | **-** | **-/+** | **+** | **-** | **-** |
| Eglence, 2013 (Turkey) | **-/+** | **+** | **+** | **-** | **+** | **+** | **-/+** |
| Elahi, 2022 (Iran) | **-** | **+** | **+** | **-/+** | **+** | **+** | **-/+** |
| Eroglu, 2022 (Turkey) | **+** | **+** | **+** | **-** | **+** | **+** | **-/+** |
| Farragher, 2021 (Canada) | **-** | **+** | **+** | **-/+** | **+** | **-** | **-** |
| Ghozhdi, 2023 (Iran) | **+** | **+** | **+** | **-** | **+** | **+** | **-/+** |
| Grigoriou, 2021 (Greece) | **-/+** | **-/+** | **-** | **-** | **+** | **+** | **-** |
| Gross, 2017 (USA) | **-** | **+** | **+** | **-** | **+** | **-/+** | **-** |
| Güler, 2023 (Turkey) | **-/+** | **+** | **-** | **-** | **+** | **+** | **-** |
| Habibzadeh, 2020 (Iran) | **-/+** | **+** | **+** | **-/+** | **+** | **+** | **+** |
| Hadadian, 2016 (Iran) | **-/+** | **+** | **+** | **-** | **-** | **-** | **-** |
| Hadadian, 2018 (Iran) | **-/+** | **+** | **+** | **-/+** | **+** | **+** | **+** |
| Haghi, 2019 (Iran) | **-/+** | **-/+** | **-** | **-** | **-** | **-** | **-** |
| Hassanzadeh, 2018 (Iran) | **-/+** | **+** | **+** | **-** | **-** | **+** | **-** |
| Huang, 2021 (Taiwan) | **-/+** | **+** | **+** | **-/+** | **-** | **+** | **-/+** |
| İlter, 2023 (Turkey) | **+** | **+** | **-** | **-** | **+** | **+** | **-** |
| Jhamb, 2023a (USA) | **-/+** | **-/+** | **-** | **-** | **+** | **-** | **-** |
| Jhamb, 2023b (USA) | **-** | **+** | **+** | **-/+** | **+** | **-/+** | **-/+** |
| Kang, 2021 (South Korea) | **-/+** | **+** | **+** | **-** | **+** | **+** | **-/+** |
| Kao, 2012 (Taiwan) | **-/+** | **+** | **+** | **-/+** | **-** | **+** | **-/+** |
| Kaplan Serin, 2020 (Turkey) | **-/+** | **+** | **+** | **-** | **+** | **+** | **-/+** |
| Karadag, 2019 (Turkey) | **-/+** | **+** | **-** | **-** | **+** | **+** | **-** |
| Kesik, 2023 (Turkey) | **-/+** | **+** | **+** | **-** | **+** | **+** | **-/+** |
| Komariah, 2022 (Indonesia) | **-/+** | **+** | **-** | **-/+** | **+** | **-** | **-** |
| Kumar, 2022 (Pakistan) | **-** | **+** | **-** | **-** | **+** | **+** | **-** |
| Lazarus, 2021 (India) | **-/+** | **+** | **+** | **-** | **-** | **-** | **-** |
| Malagoni, 2008 (Italy) | **-/+** | **+** | **+** | **-** | **+** | **-/+** | **-/+** |
| Maniam, 2014 (Malaysia) | **-/+** | **+** | **+** | **-/+** | **+** | **-** | **-/+** |
| Motedayen, 2014 (Iran) | **-/+** | **+** | **+** | **-** | **+** | **-/+** | **-/+** |
| Muz, 2017 (Turkey) | **-/+** | **-/+** | **-** | **-** | **+** | **+** | **-** |
| Neethu, 2018 (India) | **-/+** | **+** | **+** | **-** | **+** | **-** | **-** |
| Ozdemir, 2013 (Turkey) | **+** | **+** | **-** | **-** | **+** | **+** | **-** |
| Palar, 2022 (India) | **-/+** | **+** | **-** | **-/+** | **+** | **+** | **-/+** |
| Paravan, 2017 (Iran) | **-** | **+** | **+** | **-** | **+** | **-** | **-** |
| Picariello, 2021 (UK) | **+** | **+** | **+** | **+** | **+** | **+** | **+** |
| Rahimi, 2022 (Iran) | **-/+** | **+** | **+** | **-/+** | **+** | **+** | **+** |
| Rakocevic-Hrnjak, 2018 (Serbia) | **-/+** | **+** | **+** | **-** | **-** | **-** | **-** |
| Sabouhi, 2013 (Iran) | **-/+** | **+** | **+** | **-** | **+** | **-/+** | **-/+** |
| Salehi, 2020  (Iran) | **+** | **+** | **-** | **-/+** | **+** | **-/+** | **-/+** |
| Samuel Raj, 2023 (India) | **-/+** | **-/+** | **-** | **-** | **+** | **-** | **-** |
| Sari, 2023 (Indonesia) | **-/+** | **+** | **+** | **-** | **-** | **+** | **-** |
| Shahdadi, 2016 (Iran) | **-/+** | **+** | **-/+** | **-/+** | **+** | **-** | **-/+** |
| Sharifi, 2018 (Iran) | -/+ | + | + | -/+ | + | + | + |
| Suandika, 2023 (Indonesia) | -/+ | + | + | -/+ | + | + | + |
| Tsai, 2018 (Taiwan) | -/+ | + | + | + | - | -/+ | -/+ |
| Varaei, 2020  (Iran) | -/+ | + | + | - | + | + | -/+ |
| Wilkinson, 2019 (UK) | - | + | + | -/+ | + | + | -/+ |
| Wu, 2014  (Taiwan) | -/+ | + | + | - | - | + | - |
| Yeşil Bayülgen, 2023 (Turkey) | + | + | - | - | + | + | - |
| Yurtkuran, 2007 (Turkey) | + | + | + | -/+ | - | + | -/+ |
| Zeb, 2023 (Pakistan) | -/+ | -/+ | - | - | + | - | - |
| Zhou, 2023  (China) | -/+ | -/+ | - | - | + | + | - |
| Zuo, 2022  (China) | - | + | + | -/+ | + | + | -/+ |

*Note*. – = weak; -/+ = moderate; + = strong.
